# Diversity and role of plasmids in adaptation of bacteria inhabiting the Lubin copper mine in Poland, an environment rich in heavy metals

**DOI:** 10.3389/fmicb.2015.00152

**Published:** 2015-03-03

**Authors:** Lukasz Dziewit, Adam Pyzik, Magdalena Szuplewska, Renata Matlakowska, Sebastian Mielnicki, Daniel Wibberg, Andreas Schlüter, Alfred Pühler, Dariusz Bartosik

**Affiliations:** ^1^Department of Bacterial Genetics, Institute of Microbiology, Faculty of Biology, University of WarsawWarsaw, Poland; ^2^Laboratory of Environmental Pollution Analysis, Faculty of Biology, University of WarsawWarsaw, Poland; ^3^Institute for Genome Research and Systems Biology, Center for Biotechnology (CeBiTec), Bielefeld UniversityBielefeld, Germany

**Keywords:** plasmid, Tn*3* transposon, underground copper mine, terrestrial deep subsurface, extremophilic bacteria, heavy metal

## Abstract

The Lubin underground mine, is one of three mining divisions in the Lubin-Glogow Copper District in Lower Silesia province (Poland). It is the source of polymetallic ore that is rich in copper, silver and several heavy metals. Black shale is also significantly enriched in fossil organic matter in the form of long-chain hydrocarbons, polycyclic aromatic hydrocarbons, organic acids, esters, thiophenes and metalloporphyrins. Biological analyses have revealed that this environment is inhabited by extremophilic bacteria and fungi. Kupfershiefer black shale and samples of water, bottom and mineral sediments from the underground (below 600 m) Lubin mine were taken and 20 bacterial strains were isolated and characterized. All exhibited multi-resistant and hypertolerant phenotypes to heavy metals. We analyzed the plasmidome of these strains in order to evaluate the diversity and role of mobile DNA in adaptation to the harsh conditions of the mine environment. Experimental and bioinformatic analyses of 11 extrachromosomal replicons were performed. Three plasmids, including a broad-host-range replicon containing a Tn*3* family transposon, carried genes conferring resistance to arsenic, cadmium, cobalt, mercury and zinc. Functional analysis revealed that the resistance modules exhibit host specificity, i.e., they may increase or decrease tolerance to toxic ions depending on the host strain. The other identified replicons showed diverse features. Among them we identified a catabolic plasmid encoding enzymes involved in the utilization of histidine and vanillate, a putative plasmid-like prophage carrying genes responsible for NAD biosynthesis, and two *repABC-type* plasmids containing virulence-associated genes. These findings provide an unique molecular insight into the pool of extrachromosomal replicons and highlight their role in the biology and adaptation of extremophilic bacteria inhabiting terrestrial deep subsurface.

## Introduction

Bacterial plasmids, as extrachromosomal mobile genetic elements, are components of many microbial genomes. They have modular structures, since it is possible to dissect them into several functional genetic modules. The plasmid backbone is composed of a set of conserved modules, coding for replication, stability and conjugal transfer functions, which are crucial for plasmid maintenance and spread. Many plasmids also carry accessory genes determining various phenotypes, e.g., antibiotics resistance and utilization of toxic compounds. Such genetic information is not crucial for host viability, but it may play an important role in the adaptation of bacteria to various environments, including terrestrial deep subsurface habitats (Sobecky and Coombs, 2009; Heuer and Smalla, 2012; Nojiri, 2013).

It was shown that one of the most frequently found phenotypic modules carried by bacterial plasmids are heavy metal resistance genes (Silver, 1996). Moreover, metal resistance genes are often co-localized on plasmids together with antibiotic resistance genes, and they are frequently present within transposable and integrative mobile elements (Rahube et al., 2014). Such plasmid-encoded heavy metal resistance systems are usually related to chromosomally-encoded determinants found in other bacteria, which exemplifies the naturally occurring gene flow (Silver, 1996).

Horizontal gene transfer is a major mechanism contributing to bacterial diversification and adaptation, and plasmids are the main players in this process (Heuer and Smalla, 2012). Many plasmids are self-transmissible or mobilizable replicons, which can be transferred from one host to another, together with various “passenger” genes (often embedded within transposons), which can be useful under particular selection pressures (Tamminen et al., 2012). This phenomenon is extremely profitable for bacteria and, in effect, speeds up the process of evolution. To explain the link between environmental conditions and the diversity of bacterial plasmids a complex molecular analyses or, so called, meta-analyses of plasmidomes of bacteria inhabiting various (especially unique) extremophilic environments are needed (Dziewit and Bartosik, 2014).

The Lubin underground mine, is one of the three mining divisions in the Lubin-Glogow Copper District located in the Lower Silesia province (Poland). This mine is the source of polymetallic ore that is highly rich in copper (10 wt.%) and silver (100 mg kg^−1^) (Oszczepalski, 1999). Besides these two elements As, Co, V, Ni, Pb, and Zn at high concentrations were also detected (Speczik, 1994, 1995; Oszczepalski, 1999). All mentioned elements occur in the form of sulfides and sulphosalts [e.g., bornite (Cu_5_FeS_4_), chalcopyrite (CuFeS_2_)], gersdorffite (NiAsS), nickeline (NiAs), cobaltite (CoAsS), minerals belonging to the tennantite-tetrahedrite series [(Cu, Fe)_12_As_4_S_13_—(Cu, Fe)_12_Sb_4_S_13_, sphalerite (ZnS) and galena (PbS)]. A characteristic feature of these deposits is a neutral or slightly alkaline pH reaching 8.5. Black shale is also significantly enriched in fossil organic matter (up to 30%) in the form of long-chain saturated and unsaturated hydrocarbons, polycyclic aromatic hydrocarbons, organic acids and esters, as well as thiophenes and metalloporphyrins (Sklodowska et al., 2005; Matlakowska and Sklodowska, 2011). Biological analyses have revealed that this hostile environment is inhabited by endemic and extremophilic microorganisms, including bacteria and fungi (Matlakowska and Sklodowska, 2009; Rajpert et al., 2013).

In our previous work we analyzed several strains of the genus *Pseudomonas* originating from the Lubin copper mine (Szuplewska et al., 2014). Using a trap plasmid strategy to capture functional transposable elements, we identified several insertion sequences and transposons (autonomous and non-autonomous), some of which carry predicted genetic modules of adaptive value (Szuplewska et al., 2014). In this study we extended our investigation of the mobilome of bacteria inhabiting this deep underground environment. We isolated and analyzed the plasmids of strains representing various taxonomic groups, focusing on replicons conferring heavy metal resistance. Such plasmids (and their bacterial hosts) may be exploited in industrial processes, e.g. bioleaching of metals or biormediation. Moreover, the naturally occurring plasmids are good candidates for the construction of vectors for the genetic manipulations of biotechnologically important bacteria. However, this requires an in-depth understanding of the plasmid biology.

## Materials and methods

### Bacterial strains, plasmids and culture conditions

The bacterial strains and plasmids used in this study are listed in Table S1. The strains were grown in LB (Luria-Bertani) medium (Sambrook and Russell, 2001) at 37°C (*E. coli*) or 22 and 30°C (other strains). Where necessary, the medium was supplemented with sucrose (10%) and the antibiotics, kanamycin (50–1000 μg/ml) and rifampicin (50 μg/ml).

### Sample collection and bacterial isolation

Samples of black shale, mineral sediment, bottom sediment and water, collected from various sites within the Lubin underground copper mine (below 600 m), were placed in sterile plastic tubes and held at 4°C until they were processed in the laboratory.

To isolate bacterial strains, samples of solid matter (10 g) were resuspended in 20 ml of 0.85% NaCl (pH 7.0) and shaken at 22°C for 2 h. Then a series of dilutions were prepared in saline solution and plated onto solid LB medium. Diluted water samples were plated directly onto LB medium. The plates were incubated at 22°C for 2 weeks. All operations were carried out aseptically.

### Amplification and sequencing of 16S rRNA genes

A colony PCR method was used for the amplification of 16S rRNA gene fragments (Gathogo et al., 2003). PCR was performed with the primers 27f and 1492r (Lane, 1991). The amplified 16S rDNA fragments were used as templates for DNA sequencing with an ABI Prism 377 automatic sequencer (Applied Biosystems).

### Physiological analyses of the bacterial strains

The temperature, pH and salinity tolerance of bacteria were analyzed by monitoring changes in the optical density of cultures (in comparison with non-inoculated controls) according to the procedures described previously (Dziewit et al., 2013). For the motility assay LB soft agar plates containing 0.3, 0.35, or 0.4% (w/v) agar were inoculated with the bacteria using a sterile toothpick and incubated at 30°C for 48 h.

The minimum inhibitory concentrations (MICs) of selected heavy metal ions were established. For this purpose, analytical grade salts (3CdSO_4_ × 8H_2_0; CoSO_4_ × 7H_2_0; CuSO_4_; HgCl_2_; K_2_Cr_2_O_7_; NaAsO_2_; Na_2_HAsO_4_ × 7H_2_O; NiCl_2_ × 6H_2_0; NaO_3_V; ZnSO_4_ × 7H_2_0) were used in an assay procedure described previously (Dziewit et al., 2013). Isolates that grew in the presence of at least the following metal ion concentrations were considered resistant: (i) 20 mM V^5+^, (ii) 10 mM As^5+^, (iii) 1 mM As^3+^, Cd^2+^, Co^2+^, Cu^2+^, Ni^2+^, Zn^2+^, or Cr^6+^, and (iv) 0.1 mM Hg^2+^ (Nieto et al., 1987; Abou-Shanab et al., 2007).

The ability to produce siderophores was examined by application of the modified chrome azurol S (CAS) agar plate method (Schwyn and Neilands, 1987). The plates were incubated at 30°C for 72 h in the dark and the formation of halos around colonies was recorded.

### DNA manipulations, plasmid isolation and introduction of plasmid DNA into bacterial cells

Bacterial plasmids were isolated according to the method of Birnboim and Doly (Birnboim and Doly, 1979), and when required, the DNA was further purified by CsCl-ethidium bromide gradient centrifugation (Sambrook and Russell, 2001). Plasmid DNA was also isolated using a Plasmid Mini Kit (A&A Biotechnology), Plasmid Miniprep Kit Gene Matrix (EUR_x_) and GeneJET Plasmid Miniprep Kit (Thermo SCIENTIFIC). The visualization of mega-sized replicons was achieved by in-gel lysis and DNA electrophoresis according to a method described previously (Wheatcroft et al., 1990). The common DNA manipulations were performed as described earlier (Sambrook and Russell, 2001). PCR was performed in a Mastercycler (Eppendorf) using Taq DNA polymerase (Qiagen; with supplied buffer), dNTP mixture and appropriate primer pairs (Table S1). Triparental mating was performed as described previously (Bartosik et al., 2001).

### Plasmid DNA sequencing and assembly

The complete nucleotide sequences of plasmids pLM16A1, pLM20P1-5, pLM8P1, and pLM12P1 were determined in the Laboratory of DNA Sequencing and Oligonucleotide Synthesis (oligo.pl) at the Institute of Biochemistry and Biophysics, Polish Academy of Sciences, Warsaw, Poland. Plasmids pLM19O1, pLM19O2, and pLM21S1 were sequenced at the Center for Biotechnology (CeBiTec), Bielefeld University, Germany. High-throughput sequencing of the MID-tagged shotgun plasmid-libraries was performed using a Genome Sequencer FLX system (Roche/454 Life Sciences). The GS *de novo* assembler software (Roche) was applied for the sequence assemblies. Primer walking and polymerase chain reaction (PCR) were used to close physical gaps between assembled contigs. The amplified DNA fragments were sequenced using an ABI3730xl DNA Analyzer (Applied Biosystems).

### Identification of active transposable elements (TEs)

The identification of functional TEs using trap plasmid pMAT1 was performed as described previously (Szuplewska and Bartosik, 2009; Dziewit et al., 2012).

### Phage induction

Phage of *Sinorhizobium* sp. LM21 was induced using mitomycin C as described previously (Dziewit et al., 2014b).

### Functional analysis of the heavy metal resistance modules

A restriction fragment or PCR-amplified DNA regions containing the resistance genes of plasmids pLM20P1 (ARS module), pLM20P2 (CZC) and pLM16A1 (MER) were cloned into the broad-host-range mobilizable vector pBBR1MCS-2, to produce plasmids pBBR-CZCLM20, pBBR-ARSLM20 and pBBR-MERLM16 (Table S1). For comparison, two resistance cassettes of functional transposable elements identified within *Pseudomonas* spp. strains LM7 and LM14 (Szuplewska et al., 2014) were also included. Those were (i) the CZC module of insertion sequence IS*Ppu12*a (3372 bp, IS*L3* family), encoding a predicted CzcD protein, possibly involved in resistance to Co^2+^, Zn^2+^, and Cd^2+^, and (ii) MER, a mercury resistance operon of the Tn*3* family transposon Tn*5563*a (6253 bp), containing three genes—*merP, merT* (encoding mercury transporters) and *merR* (encoding a transcription regulator). To analyze these modules we used two previously obtained derivatives of the pBBR1MCS-2-based broad host range mobilizable trap plasmid pMAT1, namely pMAT-ISPPU12A and pMAT-TN5563A, carrying inserted IS*Ppu12*a and Tn*5563*a, respectively (Table S1) (Szuplewska et al., 2014).

All five plasmids containing predicted resistance modules were introduced into 13 bacterial strains (LM5, LM6, LM7, LM8, LM10, LM12, LM14, LM15, LM16, LM19, LM21, LM24, and LM25), representing *Alpha*-, *Beta*- and *Gammaproteobacteria*. As controls, two laboratory strains, *Agrobacterium tumefaciens* LBA288 (type strain of *Alphaproteobacteria*) and *E. coli* TG1 (*Gammaproteobacteria*), were also selected as the hosts for the plasmids. The resistance phenotypes of the obtained transconjugants (or transformants) were then tested by determining the minimum inhibitory concentrations (MICs) for As^3+^, As^5+^, Cd^2+^, Co^2+^, Zn^2+^, and Hg^2+^ salts in liquid culture and the obtained values were compared with those of the wild-type strains.

### Plasmid host range testing

To analyze the host range of plasmids pLM16A1, pLM20P1, and pLM20P2, three mobilizable shuttle plasmids (pABW-LM20P1, pABW-LM20P2, and pABW-LM16A1) were constructed (Table S1), containing the replication modules of the resistance plasmids and an *E. coli*-specific pMB1 (ColE1-type) replication system [vector pABW1 (Bartosik et al., 1997)]. The obtained plasmids were introduced *via* conjugation into 14 strains (LM5, LM6, LM7, LM8, LM10, LM12, LM14, LM15, LM16, LM19, LM21, LM24, LM25, and LBA288). The plasmids were also introduced via transformation into *E. coli* BR825 (*Gammaproteobacteria*). Since the ColE1-type replication system is not functional in any of the recipient strains (*E. coli* BR825 carries a mutation within the DNA polymerase I gene that blocks ColE1-type replication), all functions required for replication of the shuttle plasmids in the tested hosts were provided by the REP modules of pLM20P1, pLM20P2 or pLM16A1.

### Bioinformatic analyses

Plasmid nucleotide sequences were analyzed using Clone Manager Professional, version 9.0 (Sci-Ed software), Artemis (Carver et al., 2008) and GenDB 2.0 (Meyer et al., 2003). Similarity searches were performed using the BLAST programs (Altschul et al., 1997) and the PRIAM tool (Claudel-Renard et al., 2003). Putative tRNA genes were identified with the tRNAscan-SE program (Lowe and Eddy, 1997). Comparative genomic analyses were performed with the application of the ACT: the Artemis comparison tool (Carver et al., 2008). The reference data set for the computational prediction of metabolic pathways was obtained from the MetaCyc database (Caspi et al., 2014). Phylogenetic analysis was performed using MEGA6 (Tamura et al., 2013), applying the neighbor-joining algorithm with 1000 bootstrap replicates. The initial alignment obtained with ClustalW (Chenna et al., 2003) was manually refined. For the analysis 100 homologs (best BLAST hits) of proteins ArsB, ArsC, CzcD, and MerA retrived from the UniProt database (Apweiler et al., 2004) were used.

### Nucleotide sequence accession numbers

The sequences of 16S rRNA genes determined in this study have been deposited in GenBank (NCBI), with the following accession numbers: KF769960 (strain LM16), KF769961 (LM17), KF769962 (LM18), KF769963 (LM19), KF769964 (LM20), KF769965 (LM21), KF769966 (LM22), KF769967 (LM23), KF769968 (LM24), KF769970 (LM26).

The plasmid sequences determined in this study have been deposited in GenBank (NCBI), with the following accession numbers: KM659090 (plasmid pLM16A1), KM659091 (pLM19O1), KM659092 (pLM19O2), KM659093 (pLM20P1), KM659094 (pLM20P2), KM659095 (pLM20P3), KM659096 (pLM20P4), KM659097 (pLM20P5), KM659098 (pLM21S1), KM659088 (pLM8P1) and KM659089 (pLM12P1).

## Results

### Identification and characterization of bacteria isolated from the lubin copper mine

In this study we analyzed 20 bacterial strains isolated from various samples collected from the terrestrial deep subsurface environment, namely Lubin copper mine: (i) black shale (strains LM5, LM6, LM7, LM8, LM17, LM18, LM20, LM23, LM25, LM26); (ii) water (LM10, LM12, LM24); (iii) mineral sediment (LM11, LM14, LM21) and (iv) bottom sediment (LM15, LM16, LM19, LM22). Ten of the strains (belonging to the genus *Pseudomonas* - LM5, LM6, LM7, LM8, LM10, LM11, LM12, LM14, LM15, and LM25) were identified and subjected to preliminary characterization in previous studies (Matlakowska and Sklodowska, 2009; Szuplewska et al., 2014). However, detailed physiological or genomic analyses have not been done for these *Pseudomonas* strains so far. The remaining 10 strains were classified on the basis of comparative analyses of the obtained 16S rDNA sequences applying the RDP (Ribosomal Database Project) (Cole et al., 2009) and GenBank (NCBI) databases. This analysis revealed that the majority of the uncharacterized bacterial isolates represented different classes of *Proteobacteria*: (i) 5 strains of *Alphaproteobacteria*—*Brevundimonas* sp. LM17 and LM18, *Ochrobactrum* sp. LM19, *Paracoccus yeei* LM20 and *Sinorhizobium* sp. LM21; (ii) 1 of *Betaproteobacteria*—*Achromobacter* sp. LM16; and (iii) 2 of *Gammaproteobacteria*—*Psychrobacter* sp. LM26 and *Stenotrophomonas* sp. LM24. The two remaining strains (LM22 and LM23) were identified as members of the genus *Sphingobacterium*, belonging to the *Bacteroidetes* phylum. Based on their 16S rDNA sequences three of the analyzed strains (LM7, LM10, and LM20) could be identified as *Pseudomonas mendocina* LM7, *Pseudomonas aeruginosa* LM10 and *Paracoccus yeei* LM20.

Preliminary physiological characterization of all identified bacterial strains revealed that 13 could grow at temperatures ranging from 15 to 37°C, or even 42°C (this growth pattern is typical for mesophilic bacteria), while 7 strains were psychrotrophs, able to grow at temperatures between 4 and 37°C (the optimum temperature for all strains was either 21 or 30°C) (Table S2). All strains grew in LB medium at pH values close to 7, which is characteristic for neutrophilic bacteria. Three strains (LM7, LM16, and LM19) displayed properties of facultative alkaliphiles, since they could tolerate pH values of up to 11 (Table S2) (Slonczewski et al., 2009). Salinity tolerance testing revealed that the majority (18) of the strains were non-halophilic bacteria, while LM14 and LM26 could tolerate a higher NaCl concentration (6%); thus they were classified as halotolerant (Table S2) (Larson, 1986). The motility assay revealed that 15 of the tested strains were motile (Table S2). Moreover, application of the universal chrome azurol S (CAS) agar plate assay indicated that 16 of the analyzed strains produced iron-chelating siderophores (Table S2).

### General characterization of the plasmidome of the identified bacteria

Plasmid screening of all bacterial isolates revealed the presence of 12 circular replicons ranging in size from approx. 1.7 to 120 kb. Six strains contained plasmids: *Achromobacter s*p. LM16, *Ochrobactrum* sp. LM19, *P. yeei* LM20, *Pseudomonas* sp. LM8, *Pseudomonas* sp. LM12 and *Sinorhizobium* sp. LM21. The nucleotide sequences of all but one of these replicons [pLM8P2 of *Pseudomonas* sp. LM8 was described in our previous study (Szuplewska et al., 2014)], were obtained and analyzed. This revealed that the plasmids contained from 2 to 150 predicted genes and their average GC content varied between 53.5 and 67.3%. The plasmids carried as many as 22 types of predicted genetic modules, involved in various functions. The results of the overall characterization of the plasmids are shown in Table 1. A summary of genes identified in each plasmid, including their position, the size of the putative encoded proteins and their closest homologs, is presented in Tables S3–S7.

**Table 1:** **General features of plasmids of bacteria from the Lubin mine**.

**Plasmid name**	**Bacterial strain**	**Plasmid size (bp)**	**GC content (%)**	**Number of genes**	**Average gene length (bp)**	**Percentage of coding regions**	Genetic modules^*^
pLM16A1	*Achromobacter* sp. LM16	25,026	64.2	28	817	91.7	REP, PAR, RM, MOB, TE, MER
pLM19O1	*Ochrobactrum* sp. LM19	78,679	53.5	75	806	76.9	REP, PAR, TA, TE, RT, VIR
pLM19O2	*Ochrobactrum* sp. LM19	107,804	54.7	100	870	80.7	REP, PAR, TA, TE, TRA, VIR
pLM20P1	*Paracoccus yeei* LM20	5982	62.7	9	582	87.6	REP, TA, ARS
pLM20P2	*Paracoccus yeei* LM20	6235	63.4	9	619	89.4	REP, MOB, CZC
pLM20P3	*Paracoccus yeei* LM20	7244	62.0	9	610	75.8	REP, TA, MOB, TE
pLM20P4	*Paracoccus yeei* LM20	20,746	57.8	21	784	79.4	REP, PAR, TA, MOB
pLM20P5	*Paracoccus yeei* LM20	28,489	66.2	28	940	92.3	REP, MRS, PAR, TA, ABC, ALN, HIS, HUT, LCT, MLR, VAN
pLM8P1	*Pseudomonas* sp. LM8	1679	58.1	2	651	77.5	REP
pLM12P1	*Pseudomonas* sp. LM12	5089	67.3	4	963	56.2	REP, MOB
pLM21S1	*Sinorhizobium* sp. LM21	117,539	59.5	150	685	87.4	REP, PAR, COB, NAD

* *ABC, ABC-type transporter system; ALN, allantoate amidohydrolase; ARS, arsenic resistance module; COB, cobalamine biosynthesis module; CZC, cobalt, zinc, and cadmium resistance module; HIS, histidinol-phosphate aminotransferase; HUT, histidine catabolism module; LCT, D-lactate dehydrogenase; MER, mercury resistance module; MLR, microcystin LR degradation protein; MOB, mobilization to conjugal transfer module; MRS, multimer resolution module; NAD, NAD biosynthesis module; PAR, partitioning module; REP, replication module; RM, restriction-modification system; RT, group II intron; TA, toxin-antitoxin module; TE, transposable element; TRA, conjugal transfer module; VAN, vanillate utilization module; VIR, virulence factors*.

For each plasmid, a conserved backbone, composed of the maintenance and conjugal transfer modules (including modules responsible for mobilization for conjugal transfer) was distinguished. *In silico* analysis of the plasmid-encoded accessory modules showed plasmids containing genes of direct adaptive value: (i) heavy metal resistance plasmids (pLM16A1, pLM20P1 and pLM20P2), (ii) a catabolic plasmid (pLM20P5), and other replicons, including a (iii) plasmid-like prophage (pLM21S1), (iv) putative virulence plasmids (pLM19O1 and pLM19O2) and (v) cryptic plasmids (pLM20P3, pLM20P4, pLM8P1 and pLM12P1). In our analyses we mainly focused on heavy metal resistance plasmids.

### Genomics of heavy metal resistance plasmids

**Figure 1:**
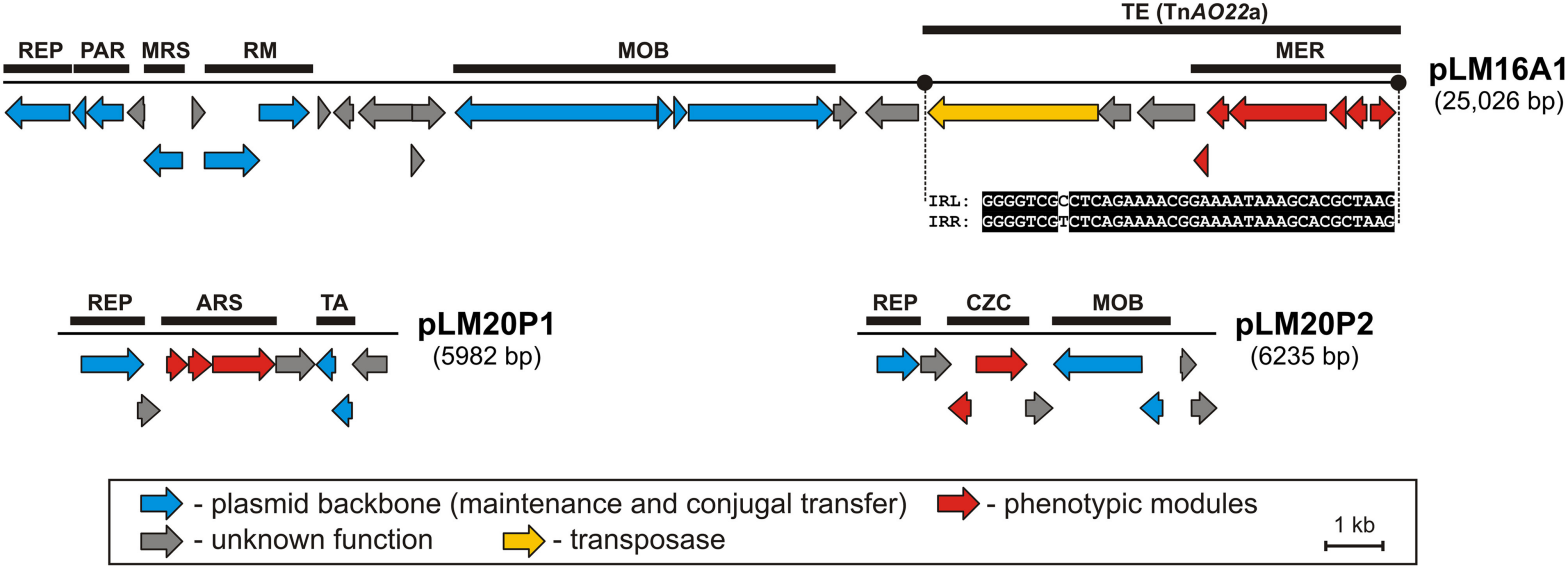
**Linear maps showing the genetic structure of the circular plasmids pLM20P1 and pLM20P2 of *P. yeei* LM20 and pLM16A1 of *Achromobacter* sp. LM16**. The predicted genetic modules are indicated by black rectangles: ARS, arsenic resistance system; CZC, cobalt, zinc and cadmium resistance system; MER, mercury resistance system; MRS, multimer resolution system; PAR, partitioning system; REP, replication system; RM, restriction-modification system; TA, toxin-antitoxin system; TE, transposon Tn*AO22*a within pLM16A1. The nucleotide sequences of the left and right terminal inverted repeats (IRL and IRR) of Tn*AO22*a are presented. Arrows indicate genes and their transcriptional orientation.

Three plasmids were found to carry predicted genetic modules involved in heavy metal resistance: pLM20P1 and pLM20P2 of *P. yeei* LM20, and pLM16A1 of *Achromobacter* sp. LM16 (Figure 1, Tables S3, S4). The average GC content of the nucleotide sequences of the plasmids is 62.7, 63.4, and 64.2% (Table 1), respectively, which is lower than the mean values determined for the total DNA of the bacteria belonging to *Achromobacter* and *Paracoccus* genus—66.4% (mean result for 11 genomes) and 67.3% (mean result for 27 genomes), respectively.

Two plasmids of *P. yeei* LM20 are small replicons (6 and 6.2 kb, respectively) encoding replication initiation proteins with homology to related proteins of plasmids occurring in several carotenoid-producing strains of *Paracoccus* spp. (Maj et al., 2013). Plasmid pLM20P1 carries three arsenic-tolerance genes (ARS module; pLM20P1_p3-pLM20P1_p5) encoding arsenate reductase (ArsC), an efflux pump (ArsB) and an ArsR family transcriptional repressor. The small cytoplasmatic arsenate reductase ArsC is responsible for the conversion of arsenate (As^5+^) into arsenite (As^3+^), while the membrane protein ArsB is an effective chemiosmotic efflux system mediating the removal of As^3+^ from the cell (Silver and Phung Le, 2005a,b). Interestingly, the ARS module of pLM20P1 shares 80% nucleotide sequence identity with the appropriate gene cluster of chromid pAMI5 of *Paracoccus aminophilus* JCM 7686 (Dziewit et al., 2014a). The second plasmid, pLM20P2 carries a CZC module, encoding a MerR family transcriptional regulator (pLM20P2_p3) and a predicted CzcD cation transport membrane protein (pLM20P2_p4) [member of the cation diffusion facilitator (CDF) protein family], which mediates cobalt (Co^2+^), zinc (Zn^2+^) and cadmium (Cd^2+^) resistance (Anton et al., 2004). This DNA region shares high level (69%) of nucleotide sequence identity with appropriate CZC module of a large, extrachromosomal, alphaproteobacterial replicon—NT26_p1 of *Rhizobium* sp. NT-26 (Andres et al., 2013).

The third plasmid, pLM16A1 of *Achromobacter* sp. LM16 (25 kb), has a mosaic structure. More than half of the plasmid genome shares at least 84% nucleotide sequence identity with transposon TNCP23 of plasmid pKLC102, coexisting as an autonomous replicon and a genomic island in *Pseudomonas aeruginosa* (Klockgether et al., 2004; Bonnin et al., 2013). Plasmid pLM16A1 contains the following modules: (i) a replication system, encoding a RepA protein (pLM16A1_p1); (ii) a partitioning system (pLM16A1_p2- pLM16A1_p3); (iii) a putative multimer resolution system (pLM16A1_p5); (iv) a type II restriction-modification system (pLM16A1_p7-pLM16A1_p8), whose restriction endonuclease (predicted recognition sequence GCCGGC) shares 68% amino acid sequence identity with R.NgoMIV from *Neisseria gonorrhoeae* MS11 (Stein et al., 1992); (v) a MOB module; and (vi) a mercury resistance module (MER; pLM16A1_p23-pLM16A1_p28) (Figure 1).

The MER module consists of 6 genes encoding proteins responsible for the enzymatic conversion of Hg^2+^ ions to the less toxic form Hg^0^ (metallic mercury). The key enzyme is the mercuric reductase (MerA), which reduces Hg^2+^ to Hg^0^. The other five MER-encoded proteins include two mercury ion transporters (MerT and MerP), two transcriptional regulators (MerR and MerD) and an accessory membrane protein (MerE) of unknown function. The pLM16A1 MER module is embedded within a transposon (Tn*AO22*a; 8240 bp), which is an isoform (99% nucleotide sequence identity) of Tn*AO22*, previously identified in *Achromobacter* sp. AO22 (Ng et al., 2009). Using a positive-selection trap vector (pMAT1) to identify functional transposable elements, we demonstrated that Tn*AO22*a is capable of transposition and therefore may contribute to the dissemination of mercury resistance.

We also performed a phylogenetic analysis of the heavy metal resistance modules of plasmids pLM20P1, pLM20P2, and pLM16A1. For the analysis ArsB, ArsC, CzcD, and MerA proteins were used. We found that the close homologs of ArsB and ArcC proteins are encoded mostly within the genomes of *Alphaproteobacteria*, including several strains of *Paracoccus* spp. (Figures S1, S2). Interestingly, proteins related to CzcD are encoded not only by *Alphaproteobacteria*, but also by members of the phylum *Thaumarchaeota* (*Archaea*), which may suggest the inter-domain gene transfer (Figure S3). The fourth analyzed protein, MerA, has close relatives encoded within the genomes of various gram-negative (*Beta-* and *Gammaproteobacteria*), as well as gram-positive bacteria (*Mycobacterium* spp. and *Bacilli*) (Figure S4).

### Host range and functional analyses of heavy metal resistance plasmids

Heavy metal tolerance of wild-type strains was determined. Ten heavy metal ions were examined: As^3+^, As^5+^, Cd^2+^, Co^2+^, Cr^6+^, Cu^2+^, Hg^2+^, Ni^2+^, V^5+^, and Zn^2+^. All tested strains showed resistance to at least 4 of the 10 tested ions. In total we found 146 (out of 200 tested) resistance phenotypes. One strain, *P. aeruginosa* LM10, exhibited resistance to all of the tested ions, while *Pseudomonas* sp. LM15 and LM25, *Achromobacter* sp. LM16, *Ochrobactrum* sp. LM19, *Sinorhizobium* sp. LM21, *Sphingobacterium* sp. LM22 and *Stenotrophomonas* sp. LM24 showed resistance to 9 of them (Table 2). All strains tolerated high levels of arsenate (60–1200 mM), nickel (1–6 mM) and copper (2–10 mM), while 19 isolates were resistant to arsenite (1–25 mM). In contrast, only 8 isolates showed (low level) resistance to chromium (VI) (Table 2).

**Table 2:** **Heavy metals resistance of bacteria isolated from the Lubin mine**.

	Heavy metals resistance (MICs) (mM)^*^									
	**As^3+^**	**As^5+^**	**Cd^2+^**	**Co^2+^**	**Cr^6+^**	**Cu^2+^**	**Hg^2+^**	**Ni^2+^**	**V^5+^**	**Zn^2+^**
*Achromobacter* sp. LM16	**25**	**1200**	**7**	**1.5**	0.8	**10**	**0.3**	**6**	**200**	**10**
*Brevundimonas* sp. LM17	**2**	**250**	0.1	0.6	0.2	**5**	0.01	**3**	1	**2**
*Brevundimonas* sp. LM18	**2**	**250**	0.1	0.7	0.4	**4**	0.02	**2**	**20**	**2**
*Ochrobactrum* sp. LM19	**9**	**1000**	**2**	**1**	**2**	**8**	0.02	**4**	**200**	**10**
*Paracoccus yeei* LM20	**6**	**400**	**1**	**1.5**	0.1	**3**	0.08	**2**	5	**1**
*Pseudomonas* sp. LM5	**1**	**400**	0.1	0.5	0.6	**3**	0.06	**2**	**75**	0.6
*Pseudomonas* sp. LM6	0.6	**75**	0.1	0.5	0.2	**3**	**0.2**	**3**	15	0.6
*Pseudomonas mendocina* LM7	**15**	**500**	0.6	0.4	**2**	**5**	**0.2**	**3**	**150**	0.9
*Pseudomonas* sp. LM8	**7**	**250**	0.1	0.5	0.3	**3**	**0.2**	**3**	15	0.4
*Pseudomonas aeruginosa* LM10	**4**	**250**	**6**	**2**	**2**	**9**	**0.2**	**3**	**30**	**15**
*Pseudomonas* sp. LM11	**3**	**500**	0.1	0.4	0.6	**2**	**0.2**	**3**	**75**	0.3
*Pseudomonas* sp. LM12	**2**	**400**	0.2	0.6	0.3	**4**	**0.2**	**3**	**75**	0.8
*Pseudomonas* sp. LM14	**15**	**250**	0.1	**1.5**	0.4	**9**	**0.1**	**4**	**250**	**6**
*Pseudomonas* sp. LM15	**7**	**500**	**1**	**4**	**3**	**8**	0.01	**6**	**40**	**6**
*Pseudomonas* sp. LM25	**6**	**500**	**4**	**3**	**2**	**8**	0.01	**6**	**30**	**5**
*Psychrobacter* sp. LM26	**4**	**200**	0.1	0.8	**2**	**5**	0.02	**1**	**100**	**2**
*Sinorhizobium* sp. LM21	**5**	**200**	**2**	**1.5**	**1**	**5**	0.02	**4**	**50**	**3**
*Sphingobacterium* sp. LM22	**3**	**250**	**2**	**2**	0.3	**7**	**0.2**	**5**	**100**	**9**
*Sphingobacterium* sp. LM23	**2**	**60**	**1**	0.3	0.2	**4**	0.02	**2**	**30**	**2**
*Stenotrophomonas* sp. LM24	**4**	**350**	**2**	0.9	**2**	**8**	**0.1**	**5**	**60**	**10**

* *MICs considered to represent the heavy metal resistance phenotype were shown in bold*.

**Figure 2:**
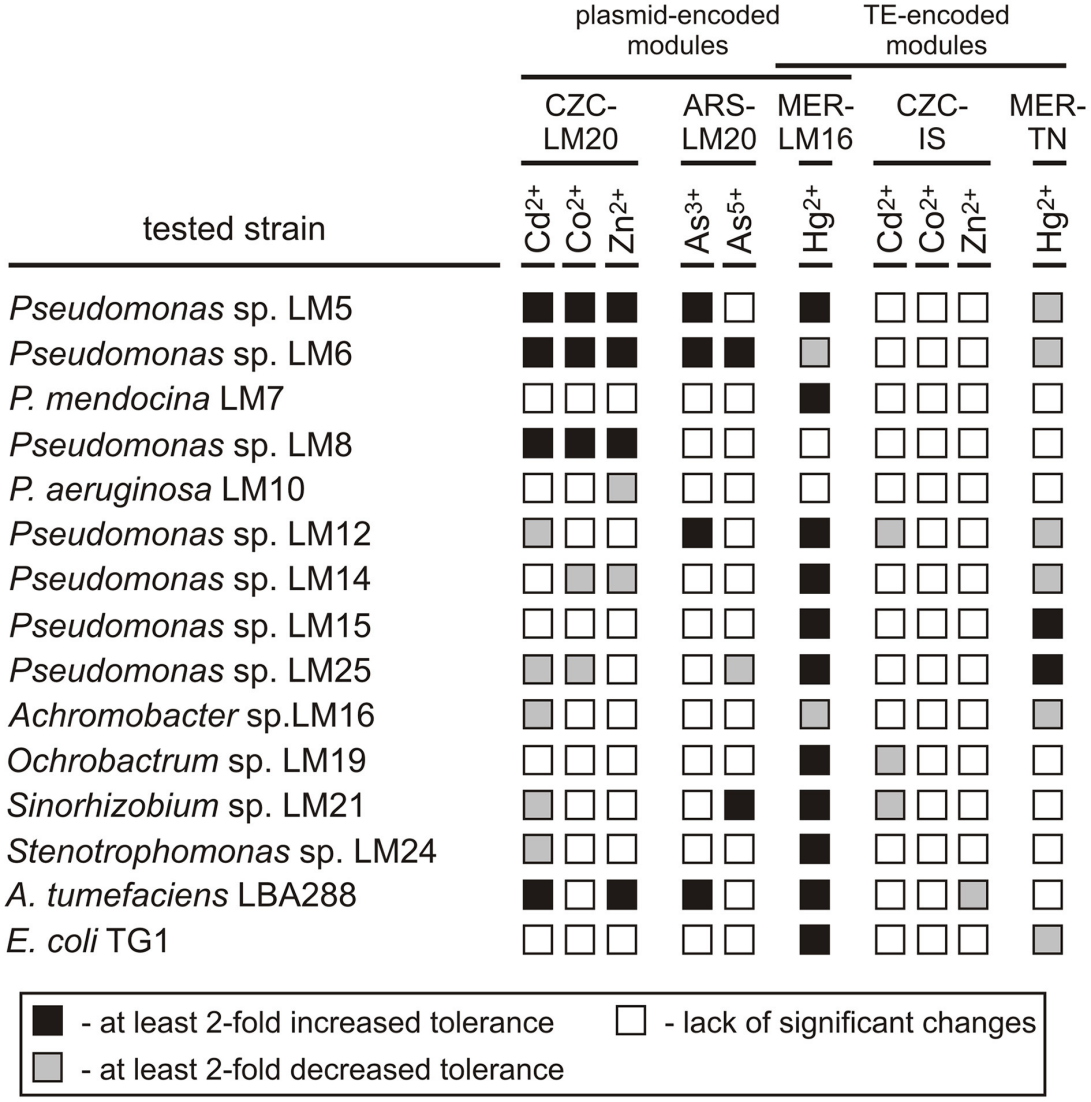
**Influence of the CZC, ARS, and MER modules on the heavy metal tolerance of bacterial strains inhabiting the Lubin copper mine, plus the controls *A. tumefaciens* and *E. coli***. CZC-LM20, cobalt, zinc and cadmium resistance module of plasmid pLM20P2; ARS-LM20, arsenic resistance module of pLM20P1; MER-LM16, mercury resistance module of plasmid pLM16A1; CZC-IS, cobalt, zinc and cadmium resistance module of IS*Ppu12*a; MER-TN, mercury resistance module of plasmid Tn*5563*a.

Then, the functionality of the resistance modules (from plasmids pLM20P1, pLM20P2, and pLM16A1, and transposable elements IS*Ppu12*a and Tn*5563*a) was tested in various hosts and the obtained values were compared with those of the wild-type strains (Figure 2). This analysis revealed that the introduction of the plasmids to the recipient strains resulted in a significant increase (at least 2-fold) in the MICs in 30 (20%) of the transconjugants, while decreases in the MICs of at least 2-fold were observed in 22 (14.7%) (Figure 2, Table S8).

The putative CZC module of plasmid pLM20P2 (CZC-LM20) was active, but produced a resistance phenotype in only a limited number of strains: *Pseudomonas* spp. LM5, LM6 and LM8 (increased resistance to Cd^2+^, Co^2+^, Zn^2+^; MIC increases of at least 3-fold) and *A. tumefaciens* LBA288 (elevated resistance to Cd^2+^ and Zn^2+^; MIC increases of 2- and 2.5-fold, respectively). In contrast, the CZC-IS module of IS*Ppu12*a did not confer resistance to Cd^2+^, Co^2+^, or Zn^2+^ in any of the tested transconjugant strains and hence probably was inactive. In a few cases, presence of the CZC-LM20 and CZC-IS modules resulted in a decrease in the MIC values (Figure 2, Table S8).

The arsenic resistance module of pLM20P1 (ARS-LM20) was tested for its ability to increase the tolerance of bacteria to arsenite and arsenate ions. Only 5 strains carrying pBBR-ARSLM20 (*Pseudomonas* spp. LM5, LM6, LM12, *Sinorhizobium* sp. LM21 and *A. tumefaciens* LBA288) exhibited at least 2-fold higher resistance to arsenite or arsenate compared to their parental wild-type strains (Figure 2, Table S8).

The MER modules analyzed in this study originated from plasmid pLM16A1 (MER-LM16 module) and transposon Tn*5563*a (MER-TN module) and were predicted to confer resistance to mercury ions. As mentioned above, the MER-LM16 module carries a complete set of genes commonly found in other well defined mercury resistance operons (Silver and Phung Le, 2005b), while MER-TN is a partial module, carrying only three genes (*merRTP*). Introduction of the MER-TN module resulted in a significant increase in the MIC for Hg^2+^ in only two strains, while the MICs of six other strains were at least 2-fold decreased. In contrast, presence of the MER-LM16 module increased tolerance to Hg^2+^ in 11 (73.3%) strains. Interestingly, introduction of plasmid pBBR-MERLM16 into two strains (LM6 and LM16) originally exhibiting high levels of resistance to mercury, resulted in an unexpected decrease in tolerance (Figure 2, Table S8).

We also tested the host range of plasmids pLM20P1, pLM20P2, and pLM16A1. The replication system of pLM16A1 was functional in all tested strains, which indicates its broad host range. In contrast, plasmids replication modules of pLM20P1 and pLM20P2 were found to have a relatively narrow host range, limited to a few strains of *Alphaproteobacteria*. Both plasmids were able to replicate in *A. tumefaciens* LBA288, and the latter one could also replicate in *Sinorhizobium* sp. LM21.

### Diversity and genomics of other plasmids of bacteria isolated from the lubin mine

**Figure 3:**
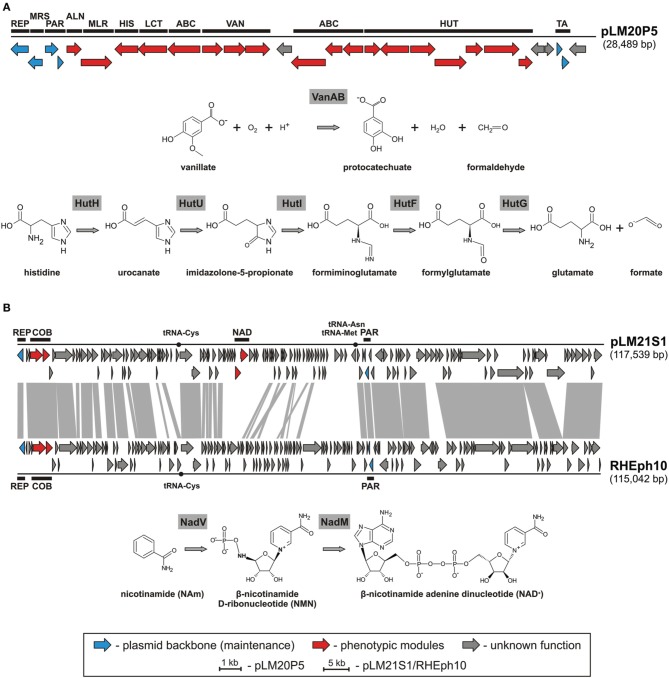
**Linear map showing the genetic structure of the circular plasmid pLM20P5 of *P. yeei* LM20 and schematic pathways for vanillate and histidine utilization (A), and linear map showing the genetic structures of the circular plasmid-like prophage pLM21S1 of *Sinorhizobium* sp. LM21 and *Rhizobium* phage RHEph10, and a schematic pathway for NAD biosynthesis (B)**. The predicted genetic modules are indicated by black rectangles: ABC, ABC-type transporter system; ALN, allantoate amidohydrolase; COB, part of a cobalamine biosynthesis module; HIS, histidinol-phosphate aminotransferase; HUT, histidine utilization system; LCT, *D*-lactate dehydrogenase; MLR, microcystin LR degradation protein; MRS, multimer resolution system; NAD, NAD^+^ biosynthesis module; PAR, partitioning system; REP, replication system; TA, toxin-antitoxin system; VAN, vanillate utilization system. Arrows indicate genes and their transcriptional orientation. The tRNA-encoding sequences are marked by black dots. The gray-shaded area connects genes of plasmid pLM21S1 and phage RHEph10 that encode homologous proteins.

Amongst identified replicons we found one catabolic plasmid, pLM20P5 of *P. yeei* LM20. The plasmid carries 28 predicted genes (Figure 3A). Of these, 12 encode enzymes directly linked with: (i) purine metabolism, (ii) pyruvate metabolism, (iii) vanillate utilization and (iv) amino acid and peptide transport and metabolism (Figure 3A, Table S3). Two of the pLM20P5-encoded phenotypic modules are of particular interest. One, responsible for the utilization of vanillate (an important intermediate in the lignin degradation process), is composed of three genes encoding vanillate *O*-demethylase [VanA and VanB subunits; (EC 1.14.13.82)] and a predicted LysR family transcriptional regulator (VanR). The formaldehyde-producing monooxygenase-type vanillate *O*-demethylase VanAB is crucial for the utilization of vanillate by its demethylation to protocatechuate (Figure 3A), which can then be degraded to central metabolism intermediates using the *meta* or *ortho* cleavage pathways (Masai et al., 2007; Chen et al., 2012). The second distinguished module (HUT) is involved in histidine utilization. It is composed of 7 genes encoding histidine ammonia lyase (HutH, EC 4.3.1.3), urocanase (HutU, EC 4.2.1.49), imidazolone propionate amidohydrolase (HutI, EC 3.5.2.7), formiminoglutamate deiminase (HutF, EC 3.5.3.13), formylglutamate amidohydrolase (HutG, EC 3.5.1.68), plus a histidine utilization repressor (HutR) and a HutD-family protein. The final products of this utilization pathway are L-glutamate and formate (Figure 3A) (Bender, 2012).

Another plasmid, pLM21S1 of *Sinorhizobium* sp. LM21, was found to carry numerous phage-related genes, encoding proteins required for DNA packaging, capsid and tail assembly, and cell lysis. This predicted plasmid-like prophage lacks an integrase gene, which suggests that it is not able to integrate into chromosomal DNA. Interestingly, pLM21S1 carries a RepC-like replication system, commonly found within large alphaproteobacterial plasmids (Cevallos et al., 2008). To check whether pLM21S1 is an active phage, we treated cells of *Sinorhizobium* sp. LM21 with mitomycin C, a classical inducer of lambdoid prophages. This approach did not cause phage induction, although it resulted in the induction of another indigenous prophage residing in the bacterial genome (Dziewit et al., 2014b). We speculate that pLM21S1 may be an inactive prophage, or alternatively it may require specific, as yet unidentified, environmental factors for induction.

Plasmid/phage pLM21S1 carries 147 putative genes and 3 tRNA-encoding sequences (Table S5). In total 83 of its genes are conserved in the genome of a related phage, RHEph10 of *Rhizobium etli* CFN42 (Santamaria et al., 2014) (Figure 3B). These two replicons carry homologous replication and partitioning systems, as well as putative genetic modules for cobalamine biosynthesis (*cobTS*) (Figure 3B). The CobT and CobS proteins form a complex, which together with an additional subunit, CobN (not encoded by the prophages) catalyzes cobalt incorporation into the corrin ring during the biosynthesis of coenzyme B12 (Debussche et al., 1992). Interestingly, pLM21S1 carries also genes involved in nicotinamide adenine dinucleotide (NAD) biosynthesis, which are not present in RHEph10. Of these, pLM21S1_p57 encodes a bifunctional nicotinamide mononucleotide adenylyltransferase/ADP-ribose pyrophosphatase (NadM, EC 2.7.7.1), while pLM21S1_p58 encodes nicotinamide phosphoribosyltransferase (NadV, EC 2.4.2.12). NadV catalyzes the transformation of nicotinamide (NAm) to β-nicotinamide ribonucleotide (NMN), which is then converted to NAD by the NadM enzyme (Figure 3B) (Martin et al., 2001). It has been suggested that the *nadV-nadM* module is not only responsible for NAD biosynthesis, but also for overall recycling of endogenous nicotinamide, which may be generated by the hydrolysis of NAD (Gerdes et al., 2006).

Next two replicons identified in the course of this study originated from *Ochrobactrum* sp. LM19. The plasmids pLM19O1 and pLM19O2 contain *repABC* modules (replication and partitioning functions) and carry 75 and 100 genes, respectively (Figure S5, Table S6). The bioinformatic analyses revealed that both replicons encode several virulence-associated proteins, including: (i) outer membrane autotransporter barrel domain-containing proteins of the type V secretion system, (ii) three invasion-associated locus B (IalB-like) proteins, whose homologs determine the erythrocyte-invasive phenotype of *Bartonella bacilliformis* (Coleman and Minnick, 2001), (iii) a YadA-like autotransporter adhesin of the non-fimbrial adhesins family, whose representatives are responsible for adhesion in infected tissues and protection against lysis (Hoiczyk et al., 2000), and (iv) an ATP-binding ABC-type transporter (HlyB) and hemolysin D (HlyD) of the hemolysin A type I secretion system (Fath and Kolter, 1993) (Figure S5, Table S6).

The remaining four plasmids (pLM20P3 and pLM20P4 of *P. yeei* LM20, pLM8P1 of *Pseudomonas* sp. LM8 and pLM12P1 of *Pseudomonas* sp. LM12) identified in bacteria from the Lubin mine were designated as cryptic replicons, since they lack accessory modules (Tables S3, S7).

## Discussion

In this study we analyzed the plasmid content of 20 bacterial strains (representing eight genera of three classes of *Proteobacteria* and one genus of the *Bacteroidetes* phylum) isolated from the Lubin underground copper mine. The plasmidome of these strains comprised 12 replicons, whose genetic structure was revealed. We demonstrated the diversity of the plasmids and defined their adaptive value, focusing on heavy metal resistance, since these toxic elements are major contaminants of the Lubin copper mine environment influencing indigenous microoganisms.

The black shale horizon is exposed to the activity of oxygen, water, and microorganisms. These factors cause the chemical and biological weathering of the rock and strongly influences the geochemical cycles of heavy metals, as well as organic carbon, which results in further redistribution of the elements within the environment (Matlakowska et al., 2010, 2012; Matlakowska and Sklodowska, 2011). The geochemical examination of environmental samples of water and bottom sediment used in presented study confirmed the presence of heavy metals. For example, the concentrations of Cu, Pb, Zn, As and Ni in sample of bottom sediment originated from underground pond were 6839 mg kg^−1^, 1128 mg kg^−1^, 115 mg kg^−1^, 110 mg kg^−1^ and 14 mg kg^−1^, respectively. Similarly, the mineral sediment taken from the surface of black shale contained copper at concentration 721 mg kg^−1^.

High concentrations of heavy metals act as a specific selection pressure permitting the survival of only well adapted indigenous strains expressing multi-resistant and hypertolerant phenotypes. Our analysis confirmed that all the strains analyzed in this work exhibited such characteristics. The majority of them were resistant to high levels of copper, arsenic and nickel, which correlates with the composition of Kupferschiefer black shale [35000 mg kg^−1^ for Cu, 398 mg kg^−1^ for As, 479 mg kg^−1^ for Ni (Matlakowska et al., 2012)]. We showed that some of the resistance phenotypes were confirmed by the mobile genetic elements.

Among the mobile genetic elements identified in the analyzed bacteria we found three plasmids (pLM16A1, pLM20P1, pLM20P2) and three transposable elements [Tn*AO22*a, Tn*5563*a, IS*Ppu12*a] that mediate resistance to arsenate, arsenite, cadmium, zinc, cobalt, and mercury. In-depth analyses demonstrated that the resistance phenotypes conferred by the particular elements are highly dependent on the host strain. Surprisingly, these results revealed that acquisition of a predicted resistance module is not always beneficial for the host, and may paradoxically lead to increased sensitivity. We speculate that this phenomenon may be the result of an unfavorable influence of the introduced genetic module on the overall homeostasis of the cell, e.g., by altering intracellular ion concentrations. All of the analyzed resistance modules encode influx/efflux pumps that are likely to have a relaxed substrate specificity [e.g., ArsB recognizes and functions with antimony, as well as arsenite (Silver and Phung Le, 2005a,b)], which may significantly influence ion fluxes.

An interesting example of a resistance module leading to increased sensitivity is the MER module of Tn*3* family transposon Tn*5563*a [deletion derivative of Tn*5044* of *Xanthomonas campestris*; (Kholodii et al., 2000)]. Transposon Tn*5563*a lacks *merA* (which is present in Tn*5044*), but it carries *merP* and *merT* genes encoding transporters responsible for the uptake of toxic mercuric ions. In six of the tested strains, the acquisition of this genetic module resulted in significant decreases in their MICs, which is probably the result of an increased intracellular concentration of toxic ions plus the inability of the strains to inactivate Hg^2+^ caused by the lack of MerA mercuric reductase. In contrast, increased tolerance was observed in two *Pseudomonas* spp. strains, possibly due to the presence of a chromosomal copy of the *merA* gene. Therefore it seems that the phenotypes determined by this module depend on the genetic background of the host strain.

The analyzed resistance modules are present on mobile genetic elements, so they may be readily transferred to other bacteria. We found that the replication system of the mobilizable plasmid pLM16A1 (containing MER) is functional in a broad range of hosts, which makes this replicon ideal for the horizontal dissemination of the mercury resistance phenotype. Moreover, the MER module of pLM16A1 (determining resistance in the great majority of tested strains) is located within an active Tn*3* family transposon (Tn*AO22*a), which enables its spread among different replicons co-residing in a single cell.

The host range of the tested plasmids correlates with the results of phylogenetic analyses, which showed that the close homologs of ArsB, ArcC, and CzcD proteins (whose genes are located within narrow host range plasmids, pLM20P1 and pLM20P2) are encoded mostly within the genomes of *Alphaproteobacteria*, while MerA protein (encoded by the broad host range plasmid pLM16A1) is conserved among various gram-negative and gram-positive bacteria. Interestingly, it was also found that the homologs of the MER module are frequently co-localized with antibiotic resistance genes in various plasmids of *Gammaproteobacteria* [e.g., pACM1 (Preston et al., 2014), pHCM1 (Parkhill et al., 2001), pKOX_R1 (Huang et al., 2013)]. Those findings reflect the pivotal role of broad host range replicons in the dissemination of various resistance genes.

In the course of this study other replicons which may contribute to adaptation of their bacterial hosts to harsh environmental conditions of the Lubin mine were also identified. Amongst them, there was a catabolic replicon pLM20P5 of *P. yeei* LM20. It carries genetic modules, enabling the utilization of histidine and vanillate. These substrates can be used as alternative sources of carbon, nitrogen and energy. Since there is a limited amount of easily degradable carbon sources in the Lubin mine, it is likely that acquisition of catabolic plasmids such as pLM20P5 may be beneficial to the host.

An intriguing finding was also the identification of an unusual plasmid-like prophage element in *Sinorhizobium* sp. LM21. The replicon pLM21S1 has features typical of both, plasmids (replication and stable maintenance modules) and phages (complete set of proteins necessary for the phage “life cycle”). Plasmid pLM21S1 is related to the phage RHEph10, which is able to infect various *Rhizbium etli* strains (Santamaria et al., 2014). Accordingly, both replicons may be considered as the archetypes of a novel group of plasmid-like prophages. Interestingly, pLM21S1 carries the *nadV-nadM* genes responsible for NAD biosynthesis. The introduction of such genes into bacteria that neither possess the NAD *de novo* biosynthesis pathway, nor the NAD salvage pathway (e.g., *Haemophilus influenzae*), may transform them into “NAD-independent” strains, able to synthesize NAD from nicotinamide (Martin et al., 2001). We postulate that the presence of pLM21S1 may be beneficial to the host because NAD production is likely to improve the overall fitness of the bacterium, since NAD (and its derivative, NADP) is the most important coenzyme in cellular redox reactions (Martin et al., 2001).

Current knowledge concerning the direction, frequency and range of DNA transfer among microorganisms living in extreme environments, and especially deep terrestrial habitats, is sparse. We believe that the approach followed in the present study, linking geochemical data with physiological characterization of bacteria and detailed analyses of their plasmidome, may considerably increase our understanding of the influence of mobile DNA and horizontal gene transfer on the biology of extremophilic bacteria.

### Conflict of interest statement

The authors declare that the research was conducted in the absence of any commercial or financial relationships that could be construed as a potential conflict of interest.

## Supplementary material

Figure S1 **Phylogenetic tree of the ArsB proteins homologous to ArsB efflux pomp encoded within pLM20P1**. The analysis was based on 100 sequences retrieved from the UniProt database. The unrooted tree was constructed using the neighbor-joining algorithm and statistical support for the internal nodes was determined by 1000 bootstrap replicates. Values of ≥50% are shown. UniProt accession numbers of the protein sequences used for the phylogenetic analysis are given in parentheses. The underlined accession numbers indicate *Paracoccus* proteins.

Figure S2 **Phylogenetic tree of the ArsC proteins homologous to ArsC arsenate reductase encoded within pLM20P1**. The analysis was based on 100 sequences retrieved from the UniProt database. The unrooted tree was constructed using the neighbor-joining algorithm and statistical support for the internal nodes was determined by 1000 bootstrap replicates. Values of ≥50% are shown. UniProt accession numbers of the protein sequences used for the phylogenetic analysis are given in parentheses. The underlined accession numbers indicate *Paracoccus* proteins.

Figure S3 **Phylogenetic tree of the CzcD proteins homologous to CzcD cation transport membrane protein encoded within pLM20P2**. The analysis was based on 100 sequences retrieved from the UniProt database. The unrooted tree was constructed using the neighbor-joining algorithm and statistical support for the internal nodes was determined by 1000 bootstrap replicates. Values of ≥50% are shown. UniProt accession numbers of the protein sequences used for the phylogenetic analysis are given in parentheses. The underlined accession numbers indicate *Paracoccus* proteins.

Figure S4 **Phylogenetic tree of the MerA proteins homologous to MerA mercuric reductase encoded within pLM16A1**. The analysis was based on 100 sequences retrieved from the UniProt database. The unrooted tree was constructed using the neighbor-joining algorithm and statistical support for the internal nodes was determined by 1000 bootstrap replicates. Values of ≥50% are shown. UniProt accession numbers of the protein sequences used for the phylogenetic analysis are given in parentheses. The underlined accession numbers indicate *Achromobacter* proteins.

Figure S5 **Linear map showing the genetic structure of the circular plasmids pLM19O1 and pLM19O2 of *Ochrobactrum* sp. LM19**. The predicted genetic modules are indicated by black rectangles: PAR, partitioning system; REP, replication system; RT, group II intron; TA, toxin-antitoxin system; TE, transposable element; TRA, conjugal transfer system; VIR, virulence module. Arrows indicate genes and their transcriptional orientation. The gray-shaded area indicates the inversion region within pLM19O1.
