# Supplementary figures and images for: Diversity and role of plasmids in adaptation of bacteria inhabiting the Lubin copper mine in Poland, an environment rich in heavy metals
Source: Front Microbiol. 2015 Mar 3;6:152. doi: 10.3389/fmicb.2015.00152 (PMC4447125; doi:10.3389/fmicb.2015.00152)

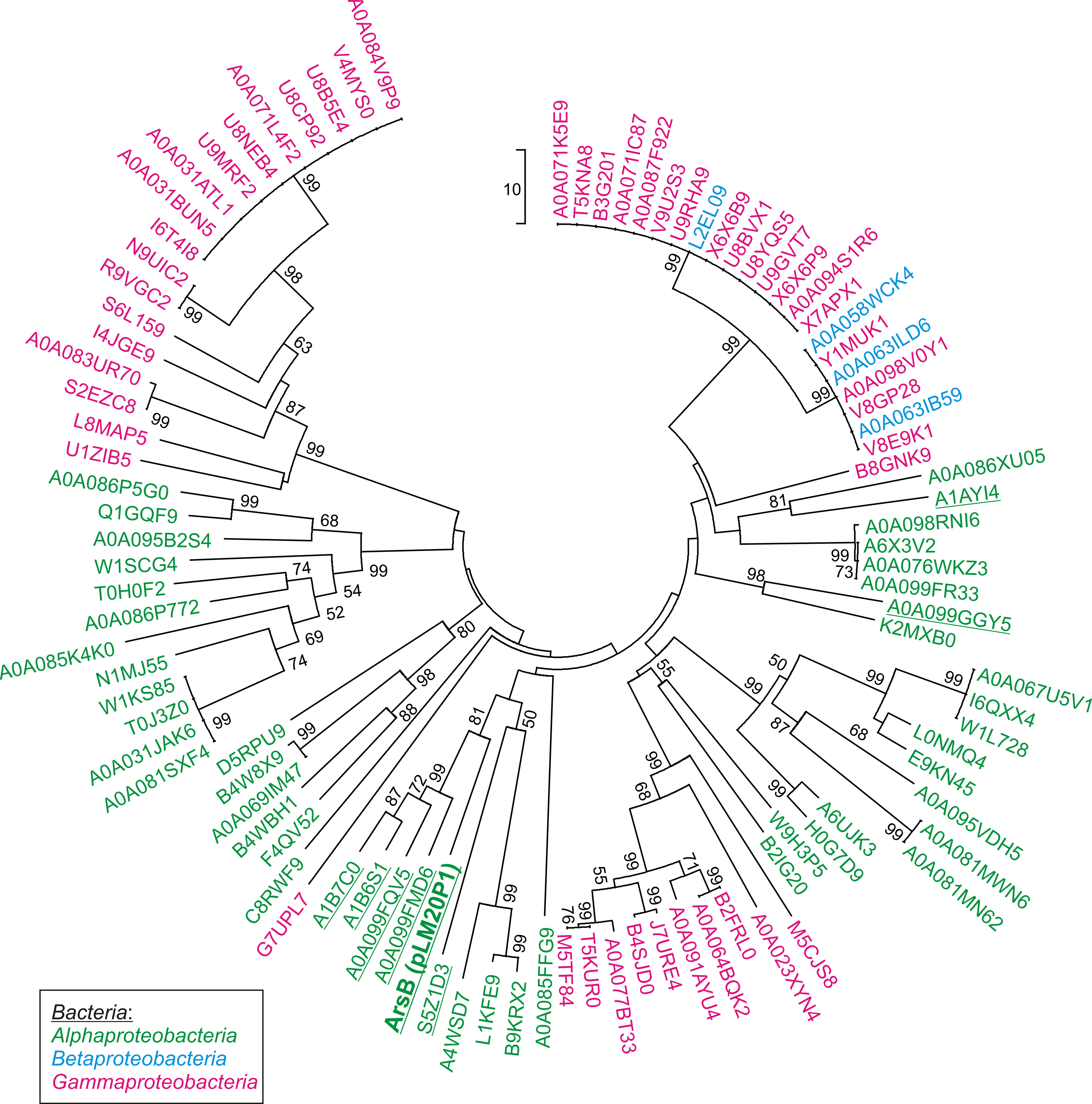

Supplement: Figure S1 — Phylogenetic tree of the ArsB proteins homologous to ArsB efflux pomp encoded within pLM20P1. The analysis was based on 100 sequences retrieved from the UniProt database. The unrooted tree was constructed using the neighbor-joining algorithm and statistical support for the internal nodes was determined by 1000 bootstrap replicates. Values of ≥50% are shown. UniProt accession numbers of the protein sequences used for the phylogenetic analysis are given in parentheses. The underlined accession numbers indicate Paracoccus proteins. [file Image1.TIF]

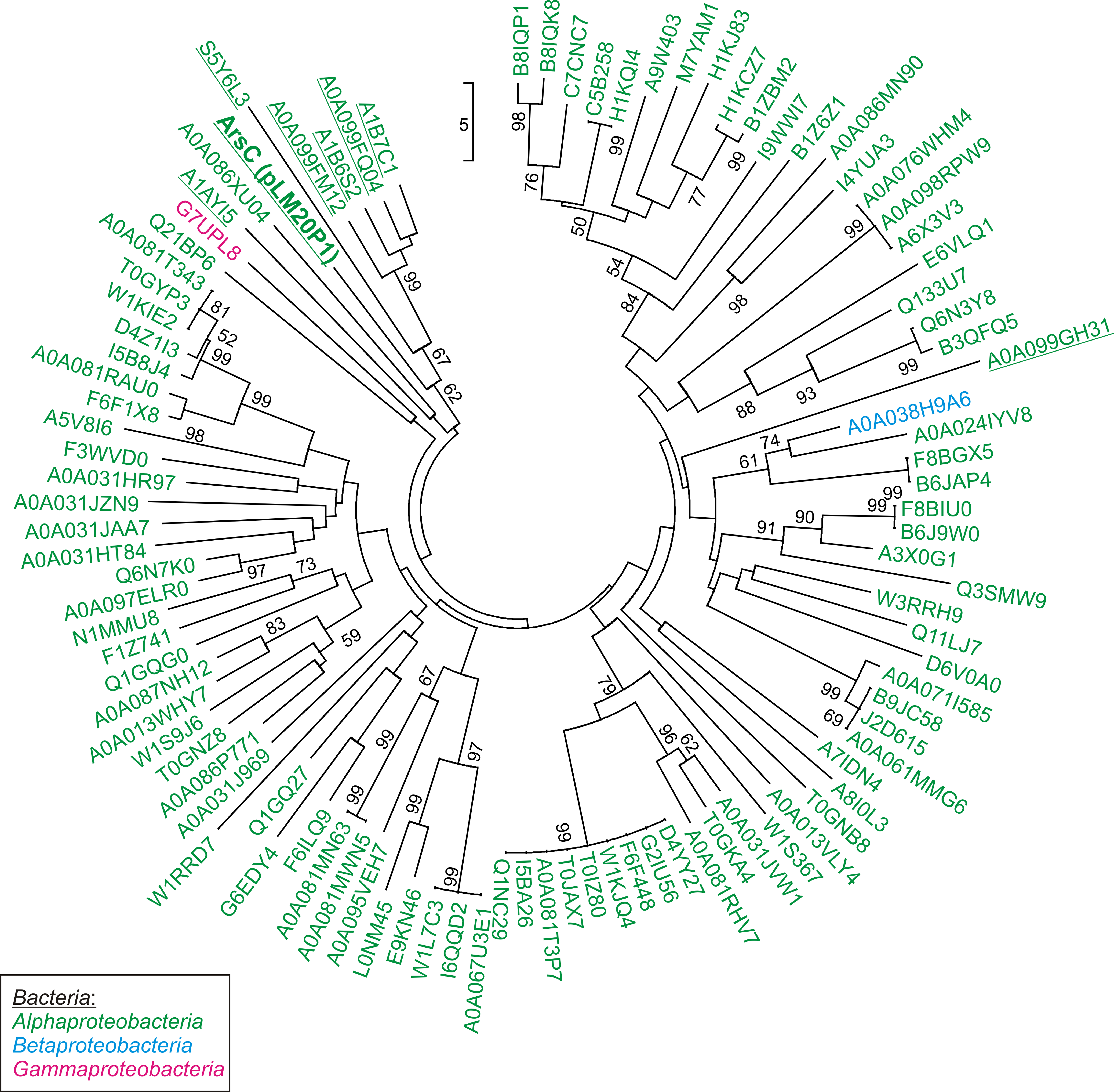

Supplement: Figure S2 — Phylogenetic tree of the ArsC proteins homologous to ArsC arsenate reductase encoded within pLM20P1. The analysis was based on 100 sequences retrieved from the UniProt database. The unrooted tree was constructed using the neighbor-joining algorithm and statistical support for the internal nodes was determined by 1000 bootstrap replicates. Values of ≥50% are shown. UniProt accession numbers of the protein sequences used for the phylogenetic analysis are given in parentheses. The underlined accession numbers indicate Paracoccus proteins. [file Image2.TIF]

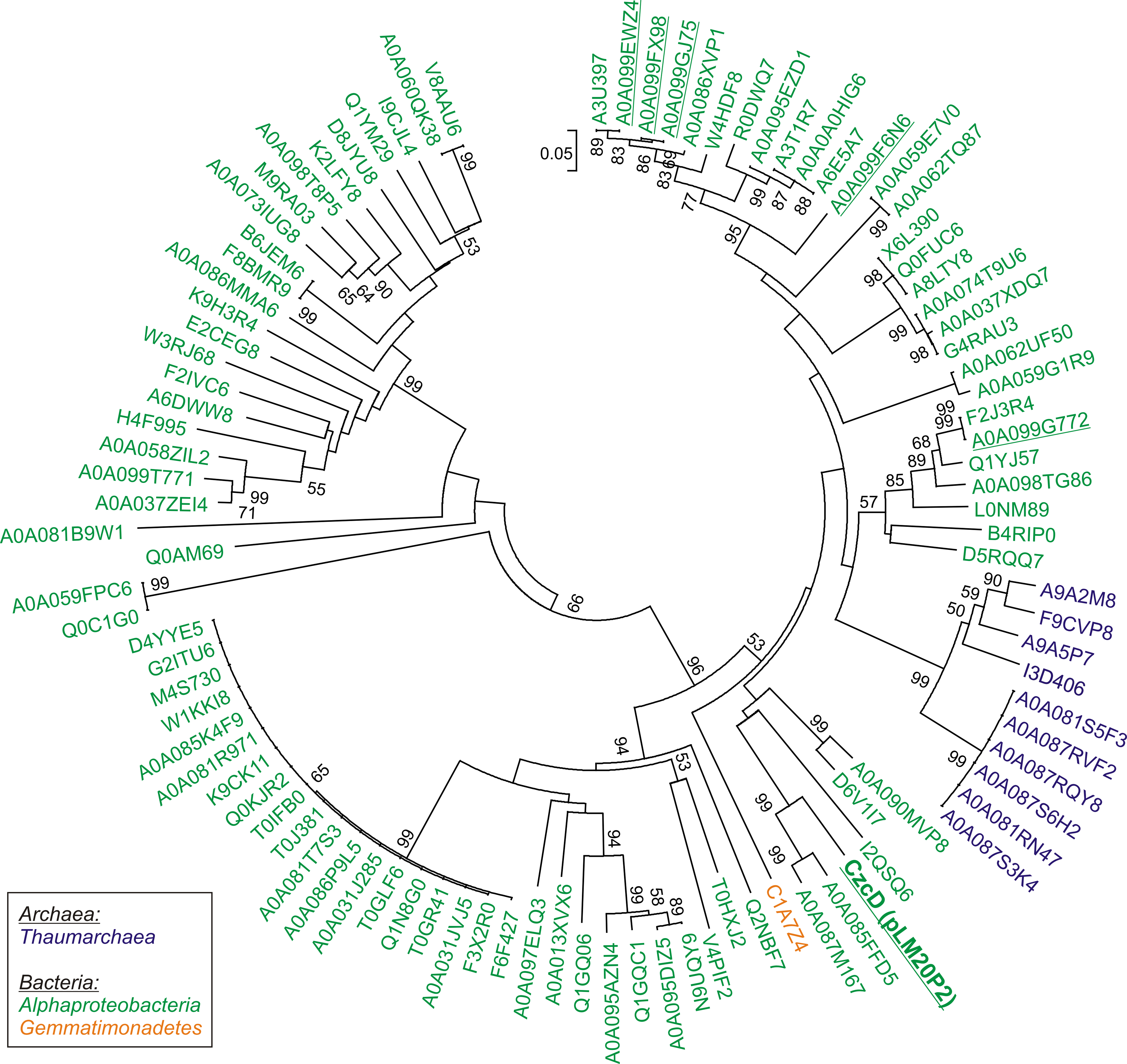

Supplement: Figure S3 — Phylogenetic tree of the CzcD proteins homologous to CzcD cation transport membrane protein encoded within pLM20P2. The analysis was based on 100 sequences retrieved from the UniProt database. The unrooted tree was constructed using the neighbor-joining algorithm and statistical support for the internal nodes was determined by 1000 bootstrap replicates. Values of ≥50% are shown. UniProt accession numbers of the protein sequences used for the phylogenetic analysis are given in parentheses. The underlined accession numbers indicate Paracoccus proteins. [file Image3.TIF]

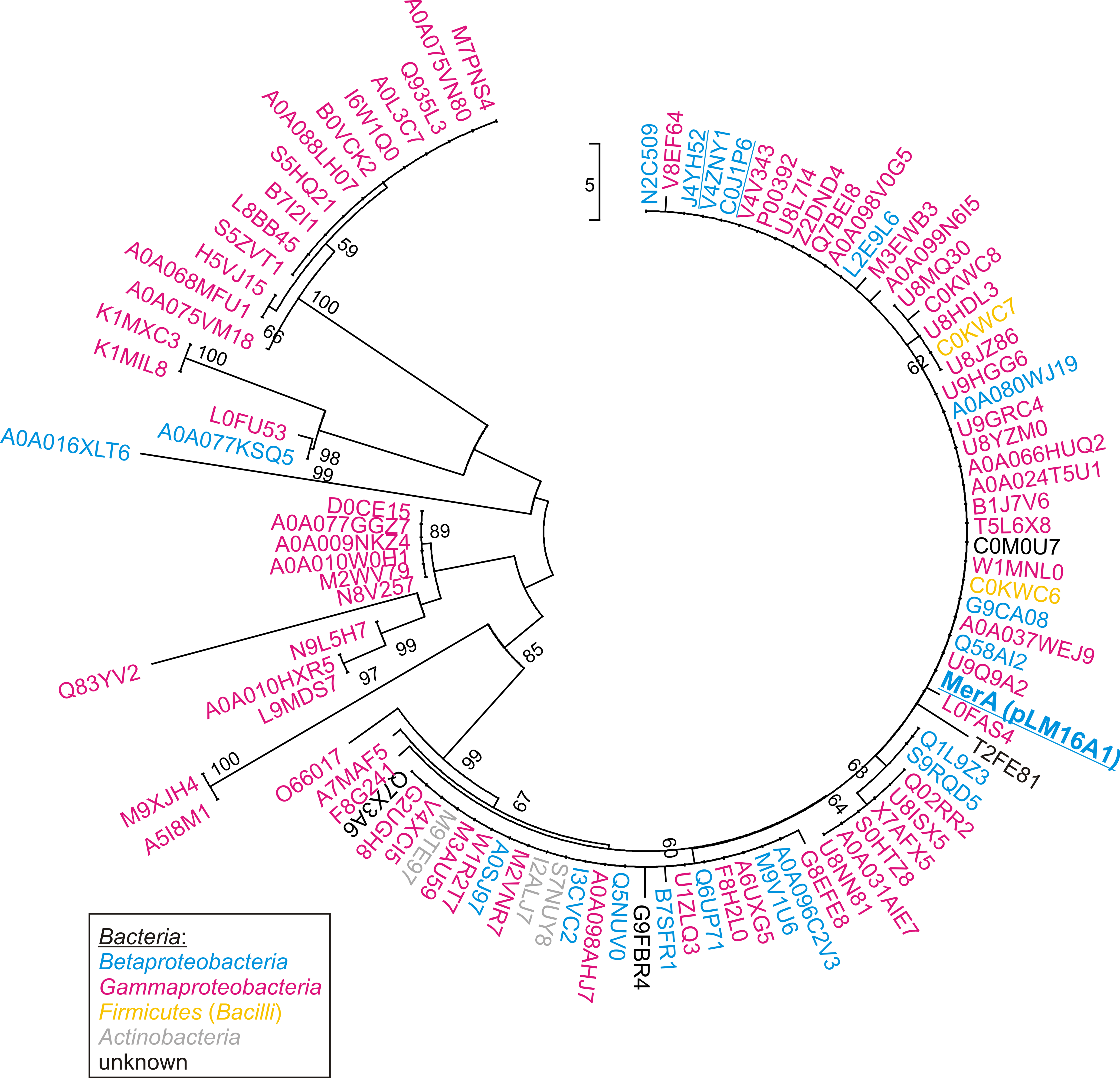

Supplement: Figure S4 — Phylogenetic tree of the MerA proteins homologous to MerA mercuric reductase encoded within pLM16A1. The analysis was based on 100 sequences retrieved from the UniProt database. The unrooted tree was constructed using the neighbor-joining algorithm and statistical support for the internal nodes was determined by 1000 bootstrap replicates. Values of ≥50% are shown. UniProt accession numbers of the protein sequences used for the phylogenetic analysis are given in parentheses. The underlined accession numbers indicate Achromobacter proteins. [file Image4.TIF]

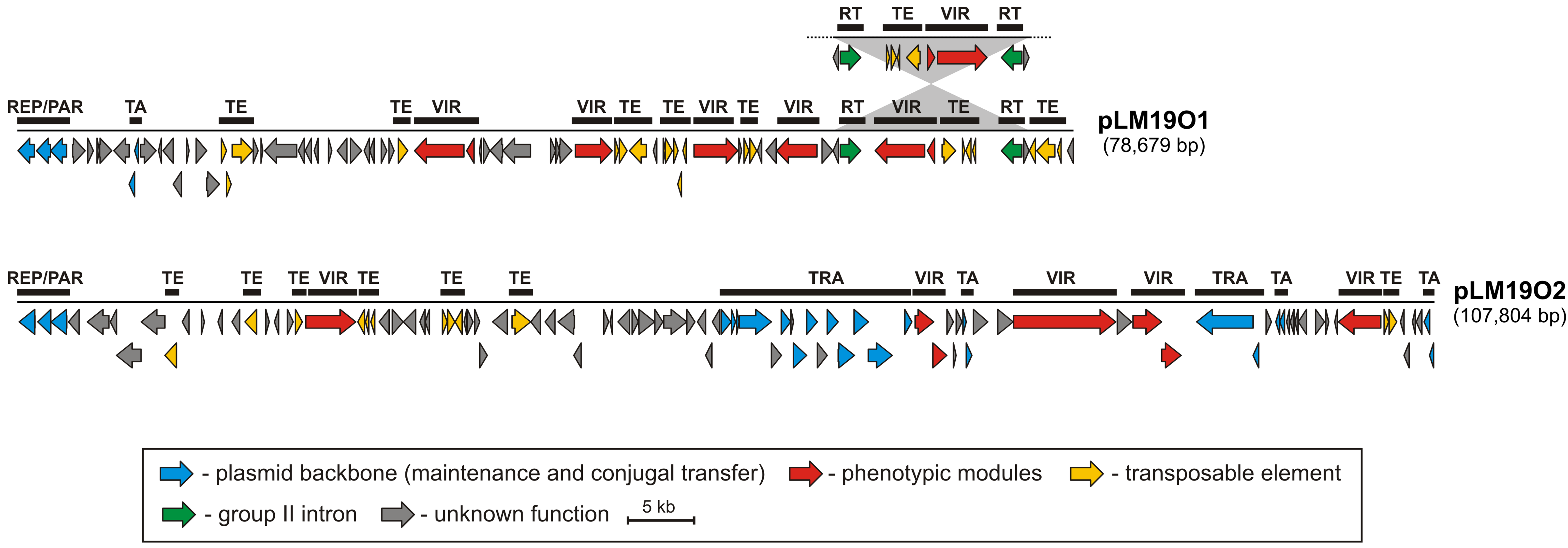

Supplement: Figure S5 — Linear map showing the genetic structure of the circular plasmids pLM19O1 and pLM19O2 of Ochrobactrum sp. LM19. The predicted genetic modules are indicated by black rectangles: PAR, partitioning system; REP, replication system; RT, group II intron; TA, toxin-antitoxin system; TE, transposable element; TRA, conjugal transfer system; VIR, virulence module. Arrows indicate genes and their transcriptional orientation. The gray-shaded area indicates the inversion region within pLM19O1. [file Image5.TIF]
