# Supplementary material for: Diversity and role of plasmids in adaptation of bacteria inhabiting the Lubin copper mine in Poland, an environment rich in heavy metals
Source: Front Microbiol. 2015 Mar 3;6:152. doi: 10.3389/fmicb.2015.00152 (PMC4447125; doi:10.3389/fmicb.2015.00152)
Supplement: Supplementary file 6 [file Table1.DOCX]

**Table S1.** Bacterial strains, plasmids and primers used in this study.

| **Strain** | | **Characteristics** | |
| --- | --- | --- | --- |
| *Achromobacter* sp. LM16 | | wild type strain from the Lubin mine | |
| *Achromobacter* sp. LM16R | | Rif^r^ derivative of the strain LM16 | |
| *Agrobacterium tumefaciens* LBA 288 | | Rif^r^ derivative | |
| *Brevundimonas* sp. LM17 | | wild type strain from the Lubin mine | |
| *Brevundimonas* sp. LM17R | | Rif^r^ derivative of the strain LM17 | |
| *Brevundimonas* sp. LM18 | | wild type strain from the Lubin mine | |
| *Brevundimonas* sp. LM18R | | Rif^r^ derivative of the strain LM18 | |
| *E. coli* BR825 | | *polA*::Tn*10 trp* | |
| *E. coli* TG1 | | F’; [*traD36 proAB+ lacIq lacZ*ΔM15] *supE44 hsdΔ5 Δ (lac- proAB)* | |
| *Ochrobactrum* sp. LM19 | | wild type strain from the Lubin mine | |
| *Ochrobactrum* sp. LM19R | | Rif^r^ derivative of the strain LM19 | |
| *Paracoccus yeei* LM20 | | wild type strain from the Lubin mine | |
| *Paracoccus yeei* LM20R | | Rif^r^ derivative of the strain LM20 | |
| *Pseudomonas* sp. LM5 | | wild type strain from the Lubin mine | |
| *Pseudomonas* sp. LM5R | | Rif^r^ derivative of the strain LM21 | |
| *Pseudomonas* sp. LM6 | | wild type strain from the Lubin mine | |
| *Pseudomonas* sp. LM6R | | Rif^r^ derivative of the strain LM6 | |
| *Pseudomonas* sp. LM7 | | wild type strain from the Lubin mine | |
| *Pseudomonas* sp. LM7R | | Rif^r^ derivative of the strain LM7 | |
| *Pseudomonas* sp. LM8 | | wild type strain from the Lubin mine | |
| *Pseudomonas* sp. LM8R | | Rif^r^ derivative of the strain LM8 | |
| *Pseudomonas* sp. LM10 | | wild type strain from the Lubin mine | |
| *Pseudomonas* sp. LM10R | | Rif^r^ derivative of the strain LM10 | |
| *Pseudomonas* sp. LM11 | | wild type strain from the Lubin mine | |
| *Pseudomonas* sp. LM11R | | Rif^r^ derivative of the strain LM11 | |
| *Pseudomonas* sp. LM12 | | wild type strain from the Lubin mine | |
| *Pseudomonas* sp. LM12R | | Rif^r^ derivative of the strain LM12 | |
| *Pseudomonas* sp. LM14 | | wild type strain from the Lubin mine | |
| *Pseudomonas* sp. LM14R | | Rif^r^ derivative of the strain LM14 | |
| *Pseudomonas* sp. LM15 | | wild type strain from the Lubin mine | |
| *Pseudomonas* sp. LM15R | | Rif^r^ derivative of the strain LM15 | |
| *Pseudomonas* sp. LM25 | | wild type strain from the Lubin mine | |
| *Pseudomonas* sp. LM25R | | Rif^r^ derivative of the strain LM25 | |
| *Psychrobacter* sp. LM26 | | wild type strain from the Lubin mine | |
| *Psychrobacter* sp. LM26R | | Rif^r^ derivative of the strain LM26 | |
| *Sinorhizobium* sp. LM21 | | wild type strain from the Lubin mine | |
| *Sinorhizobium* sp. LM21R | | Rif^r^ derivative of the strain LM21 | |
| *Sphingobacterium* sp. LM 22 | | wild type strain from the Lubin mine | |
| *Sphingobacterium* sp. LM 22R | | Rif^r^ derivative of the strain LM22 | |
| *Sphingobacterium* sp. LM23 | | wild type strain from the Lubin mine | |
| *Sphingobacterium* sp. LM23R | | Rif^r^ derivative of the strain LM23 | |
| *Stenotrophomonas* sp. LM24 | | wild type strain from the Lubin mine | |
| *Stenotrophomonas* sp. LM24R | | Rif^r^ derivative of the strain LM24 | |
| **Plasmid** | | **Characteristics** | |
| pABW1 | | Km^r^; 4.5 kb; *ori* pMB1; *oriT* RK2; *lacZα*; MCS | |
| pABW-LM16A1 | | Km^r^; 6.2 kb; pABW1 derivative carrying REP module of plasmid pLM16A1 (amplified by PCR with primers LLM16P1B and RLM16P1E) inserted between BamHI and EcoRI sites | |
| pABW-LM20P1 | | Km^r^; 6.5 kb; pABW1 derivative carrying REP module of plasmid pLM20P1 (amplified by PCR with primers LLM20P1E and RLM20P1P) inserted between EcoRI and PstI sites | |
| pABW-LM20P2 | | Km^r^; 6.3 kb; pABW1 derivative carrying REP module of plasmid pLM20P2 (amplified by PCR with primers LLM20P2E and RLM20P2B) inserted between BamHI and EcoRI sites | |
| pABW-LM21S1 | | Km^r^; 7.3 kb; pABW1 derivative carrying 2.8-kb BamHI/SacI restriction fragment of pLM21S1 (contains REP module) inserted between BamHI and SacI sites | |
| pBBR1MCS-2 | | Km^r^; 5.1 kb; *ori* pBBR1; *oriT* RK2; *lacZα*; MCS | |
| pBBR-ARSLM20 | | Km^r^; 8.0 kb; pBBR1MCS-2 derivative carrying ARS module of plasmid pLM20P1 (amplified by PCR with primers LARSLM20 and RARSLM20) inserted between HindIII and EcoRI sites | |
| pBBR-CZCLM20 | | Km^r^; 7.2 kb; pBBR1MCS-2 derivative carrying CZC module of plasmid pLM20P2 (amplified by PCR with primers LCZCLM20 and RCZCLM20) inserted between BamHI and EcoRI sites | |
| pBBR-MERLM16 | | Km^r^; 12.3 kb; pBBR1MCS-2 derivative carrying 7.2-kb DraI/KpnI restriction fragment of pLM16A1 (contains MER module) inserted between SmaI and KpnI sites | |
| pLM16A1 | | natural plasmid of *Achromobacter* sp. LM16 | |
| pLM19O1 | | natural plasmid of *Ochrobactrum* sp. LM19 | |
| pLM19O2 | | natural plasmid of *Ochrobactrum* sp. LM19 | |
| pLM20P1 | | natural plasmid of *Paracoccus yeei* LM20 | |
| pLM20P2 | | natural plasmid of *Paracoccus yeei* LM20 | |
| pLM20P3 | | natural plasmid of *Paracoccus yeei* LM20 | |
| pLM20P4 | | natural plasmid of *Paracoccus yeei* LM20 | |
| pLM20P5 | | natural plasmid of *Paracoccus yeei* LM20 | |
| pLM8P1 | | natural plasmid of *Pseudomonas* sp. LM8 | |
| pLM12P1 | | natural plasmid of *Pseudomonas* sp. LM12 | |
| pLM21S1 | | natural plasmid of *Sinorhizobium* sp. LM21 | |
| pMAT1 | | Km^r^; 7.0 kb; *ori* pBBR1; *oriT* RK2; *sacB*; entrapment vector | |
| pMAT-ISPPU12A | | Km^r^; 10.4kb; pMAT1 derivative carrying IS*PPu12a* inserted in *sacB* gene | |
| pMAT-TN5563A | | Km^r^; 13.3 kb; pMAT1 derivative carrying Tn*5563a* inserted in *sacB* gene | |
| PMAT-TNAO22A | | Km^r^; 15.2 kb; pMAT1 derivative carrying Tn*AO22a* inserted in *sacB* gene | |
| pRK2013 | | Km^r^; 48.0 kb; *ori* ColE1; Tra+ (RK2 conjugal transfer system) | |
| **Primer** | **Sequence (5’->3’)** | | **Function** |
| LARSLM20 | TCAAGCTTGAGATTGCGGCGGACCTGAA | | amplification of ARS module of pLM20P1 |
| RARSLM20 | TCGAATTCACTCGACCGGCACTCCTGAA | | amplification of ARS module of pLM20P1 |
| LCZCLM20 | GCGAATTCAGCCTCCTTCAGCGCATCCT | | amplification of CZC module of pLM20P2 |
| RCZCLM20 | TAGGATCCCTTGGTGCAGCAGGTGAACG | | amplification of CZC module of pLM20P2 |
| LLM16P1B | TAGGATCCCCTTGGCGTTCAACCTGTGG | | amplification of REP module of pLM16A1 |
| RLM16P1E | GCGAATTCAGTATGTCGCCAGTCTGCCG | | amplification of REP module of pLM16A1 |
| LLM20P1E | CGGAATTCGCAAGGCAAGATAAGGCGGC | | amplification of REP module of pLM20P1 |
| RLM20P1P | TACTGCAGGGCTTGAGGCTCATCCATCG | | amplification of REP module of pLM20P1 |
| LLM20P2E | GCGAATTCATCTATGGAACGCGCCGCTG | | amplification of REP module of pLM20P2 |
| RLM20P2B | TAGGATCCCGAATGCCGAGTGATCGAGG | | amplification of REP module of pLM20P2 |
| A289SB | CAGACCGCTAACACAGTACA | | localization of TE within *sacB* gene of pMAT1 |
| A869SB | TTAGGATCTCCGGCTAATGC | | localization of TE within *sacB* gene of pMAT1 |
| B824SB | ACTATCACGGCTACCACATC | | localization of TE within *sacB* gene of pMAT1 |
| B1253SB | TTGTCGCCTGAGCTGTAGTT | | localization of TE within *sacB* gene of pMAT1 |
| C1225SB | GATGAAGGCAACTACAGCTC | | localization of TE within *sacB* gene of pMAT1 |
| C1639SB | GACGATTGACGGCATTACGT | | localization of TE within *sacB* gene of pMAT1 |
| D1619SB | TGACGATTGACGGCATTACG | | localization of TE within *sacB* gene of pMAT1 |
| D1928SB | GACAGCATCCTTGAACAAGG | | localization of TE within *sacB* gene of pMAT1 |
| Pc2t | TCTCCGGTTTGGAGTGATTT | | identification of inversion within pLM19O1 |
| Pc4t | ACGAGCGAAACCAGCCTACATA | | identification of inversion within pLM19O1 |
| Pc5t | TTCGCGCTGCAGGCAAATA | | identification of inversion within pLM19O1 |
| P10h | AGCGGTCGAAGCCTTTCAT | | identification of inversion within pLM19O1 |
