# Supplementary material for: Diversity and role of plasmids in adaptation of bacteria inhabiting the Lubin copper mine in Poland, an environment rich in heavy metals
Source: Front Microbiol. 2015 Mar 3;6:152. doi: 10.3389/fmicb.2015.00152 (PMC4447125; doi:10.3389/fmicb.2015.00152)
Supplement: Supplementary file 7 [file Table2.DOC]

| Strain | *Pseudomonas* sp. LM5 | *Pseudomonas* sp. LM6 | *Pseudomonas* *mendocina* LM7 | *Pseudomonas* sp. LM8 | *Pseudomonas* *aeruginosa* LM10 | *Pseudomonas* sp. LM11 | *Pseudomonas* sp. LM12 | *Pseudomonas* sp. LM14 | *Pseudomonas* sp. LM15 | *Pseudomonas* sp. LM25 | *Achromobacter* sp. LM16 | *Brevundimonas* sp. LM17 | *Brevundimonas* sp. LM18 | *Ochrobactrum* sp. LM19 | *Paracoccus yeei* LM20 | *Sinorhizobium* sp. LM21 | *Sphingobacterium* sp. LM22 | *Sphingobacterium* sp. LM23 | *Stenotrophomonas* sp. LM24 | *Psychrobacter* sp. LM26 |
| --- | --- | --- | --- | --- | --- | --- | --- | --- | --- | --- | --- | --- | --- | --- | --- | --- | --- | --- | --- | --- |
| Temperature tolerance (ᵒC) | 4-37 | 15-37 | 15-37 | 15-42 | 15-42 | 4-37 | 4-37 | 4-37 | 15-37 | 15-37 | 15-42 | 4-37 | 15-30 | 15-37 | 15-37 | 15-37 | 4-37 | 15-37 | 15-37 | 4-37 |
| Optimal temperature (ᵒC) | 21 | 21 | 21 | 21 | 21 | 15 | 21 | 21 | 37 | 21 | 30 | 21 | 21 | 30 | 30 | 30 | 21 | 30 | 30 | 21 |
| pH tolerance | 6-10 | 6-9 | 5-11 | 6-9 | 5-10 | 6-8 | 6-10 | 5-10 | 5-10 | 5-10 | 5-11 | 6-8 | 6-10 | 5-11 | 4-9 | 4-9 | 5-9 | 6-9 | 5-10 | 6-10 |
| NaCl tolerance (%) | 3 | 3 | 3 | 3 | 3 | 3 | 3 | 6 | 3 | 3 | 3 | 3 | 3 | 3 | 3 | 3 | 3 | 3 | 3 | 6 |
| Motility | – | + | – | + | + | + | + | – | + | + | + | – | + | + | + | + | + | – | + | + |
| Siderophore production | + | + | + | + | + | + | + | + | + | + | + | – | – | + | – | + | + | – | + | + |

**Table S2.** Physiological characterization of bacteria isolated from the Lubin copper mine.
