# Supplementary material for: Diversity and role of plasmids in adaptation of bacteria inhabiting the Lubin copper mine in Poland, an environment rich in heavy metals
Source: Front Microbiol. 2015 Mar 3;6:152. doi: 10.3389/fmicb.2015.00152 (PMC4447125; doi:10.3389/fmicb.2015.00152)
Supplement: Supplementary file 8 [file Table3.DOC]

**Table S3.** Genes located within plasmids pLM20P1 – pLM20P5 of *Paracoccus yeei* LM20.

| Geneno. | **Coding region**  **(bp)** | **Strand** | **Protein size (aa)** | **Possible function** | **Best BLAST hits** | | |
| --- | --- | --- | --- | --- | --- | --- | --- |
|  |  |  |  |  | **% identity (aa)** | **Organism** | **GenBank accession no.** |
| **Plasmid pLM20P1 (5982 bp)** | | | | | | | |
| 1 | 408-1469 | → | 353 | replication initiator protein | 65%  (205/314) | *Paracoccus haeundaensis* LMG P-21903 (plasmid pHAE2) | YP_006963003 |
| 2 | 1381-1764 | → | 127 | MerR family transcriptional regulator | 59%  (74/125) | *P. haeundaensis* LMG P-21903 (plasmid pHAE2) | YP_006961988 |
| 3 | 1885-2244 | → | 119 | ArsR family transcriptional repressor | 80%  (94/118) | *Paracoccus aminophilus* JCM 7686 (plasmid pAMI5) | YP_008406626 |
| 4 | 2247-2672 | → | 141 | arsenate reductase, ArsC | 83%  (117/141) | *Paracoccus denitrificans* PD1222 | YP_916912 |
| 5 | 2687-3763 | → | 358 | arsenic resistance protein ArsB | 88%  (315/358) | *P. denitrificans* PD1222 | YP_916911 |
| 6 | 3760-4467 | → | 235 | NADPH-dependent FMN reductase | 91%  (213/235) | *P. denitrificans* PD1222 | YP_916910 |
| 7 | 4497-4802 | ← | 101 | antitoxin of toxin-antitoxin system | 59%  (56/95) | *Sphingomonas sp.* PAMC 26617 | WP_010165135 |
| 8 | 4789-5082 | ← | 97 | toxin of toxin-antitoxin system | 62%  (61/98) | *Photorhabdus temperate* subsp. temperata M1021 | WP_021326461 |
| 9 | 5114-5689 | ← | 191 | hypothetical protein | 45%  (87/195) | *Paracoccus aestuarii* DSM 19484 (plasmid pAES2) | YP_006961986 |
| **Plasmid pLM20P2 (6235 bp)** | | | | | | | |
| 1 | 310-1044 | → | 244 | replication initiator protein | 95%  (231/244) | *P. aestuarii* DSM 19484 (plasmid pAES4) | YP_006963008 |
| 2 | 1122-1604 | → | 160 | hypothetical protein | 73%  (80/109) | *P. aestuarii* DSM 19484 (plasmid pAES4) | YP_006963009 |
| 3 | 1539-1979 | ← | 146 | MerR family transcriptional regulator | 63%  (80/127) | *Afipia birgiae* | WP_019197536 |
| 4 | 2048-2917 | → | 289 | Co/Zn/Cd efflux system component | 63%  (180/285) | *Phenylobacterium zucineum* HLK1 (plasmid “unnamed”) | YP_002128790 |
| 5 | 2914-3393 | → | 159 | signal peptidase | 42%  (59/140) | *Hyphomonas neptunium* ATCC 15444 | YP_760434 |
| 6 | 3395-4936 | ← | 513 | mobilization protein A (MobA) | 52%  (266/514) | *Paracoccus marcusii* DSM 11574 (plasmid pMARC2) | YP_008003480 |
| 7 | 4936-5298 | ← | 120 | mobilization protein C (MobC) | 69%  (68/99) | *P. marcusii* DSM 11574 (plasmid pMARC2) | YP_008003481 |
| 8 | 5593-5841 | → | 82 | hypothetical protein | 70%  (57/82) | *Acidiphilium sp.* PM | WP_007423962 |
| 9 | 5828-6235 | → | 135 | hypothetical protein | 62%  (84/135) | *Mesorhizobium metallidurans* | WP_008878340 |
| **Plasmid pLM20P3 (7244 bp)** | | | | | | | |
| 1 | 490-1038 | → | 182 | replication initiator protein | 76%  (132/174) | *Sphingobium xenophagum* QYY | YP_195758 |
| 2 | 1100-1552 | → | 150 | hypothetical protein | 52%  (60/115) | *P. haeundaensis* LMG P-21903 (plasmid pHAE1) | YP_006961988 |
| 3 | 1864-3078 | → | 404 | transposase | 97%  (390/404) | *Maritimibacter alkaliphilus* | WP_008336078 |
| 4 | 3181-3813 | ← | 210 | hypothetical protein | 62%  (125/202) | *Burkholderia sp.* CCGE1001 | YP_004229923 |
| 5 | 4220-5437 | ← | 405 | mobilization protein A (MobA) | 78%  (324/414) | *P. aminophilus* JCM 7686 (plasmid pAMI3) | YP_003305343 |
| 6 | 5594-5860 | → | 88 | mobilization protein C (MobC) | 84%  (74/88) | *P. aminophilus* JCM 7686 (plasmid pAMI3) | YP_003305344 |
| 7 | 5857-6417 | → | 186 | hypothetical protein | 59%  (109/184) | *P. aminophilus* JCM 7686 (plasmid pAMI3) | YP_003305345 |
| 8 | 6573-6869 | ← | 98 | antitoxin of toxin-antitoxin system | 95%  (93/98) | *P. aestuarii* DSM 19484 (plasmid pAES7) | YP_006965325 |
| 9 | 6873-7169 | ← | 98 | toxin of toxin-antitoxin system | 85%  (82/98) | *P. aestuarii* DSM 19484 (plasmid pAES7) | YP_006965326 |
| **Plasmid pLM20P4 (20746 bp)** | | | | | | | |
| 1 | 738-1670 | → | 310 | replication initiator protein | 67%  (206/307) | *P. aminophilus* JCM 7686 (plasmid pAMI7) | YP_004103180 |
| 2 | 1718-2350 | → | 210 | partitioning protein ParA | 60%  (123/206) | *M. metallidurans* | WP_008878357 |
| 3 | 2357-2659 | → | 100 | hypothetical protein | 53%  (29/55) | *M. metallidurans* | WP_008878356 |
| 4 | 3029-3643 | → | 204 | partitioning protein ParA | 62%  (127/204) | *Loktanella hongkongensis* | WP_017930027 |
| 5 | 4326-5303 | → | 325 | NAD-dependent dehydratase | 61%  (183/299) | *Labrenzia aggregata* | WP_006938211 |
| 6 | 5481-5867 | → | 128 | transposase, truncated | 81%  (98/121) | *Microvirga sp.* WSM3557 | WP_009488342 |
| 7 | 5908-6996 | ← | 362 | hypothetical protein | 31%  (80/257) | *Sulfurovum sp.* NBC37-1 | YP_001359038 |
| 8 | 7169-11068 | ← | 1299 | glycosyl transferase | 56%  (738/1315) | *Ruegeria sp. PR1b* | NP_861985 |
| 9 | 11111-12592 | ← | 493 | hypothetical protein | 36%  (186/513) | *P. aminophilus* JCM 7686 (plasmid pAMI5) | YP_008406625 |
| 10 | 13026-13241 | ← | 71 | transposase, truncated | 61%  (19/31) | *Thalassospira profundimaris* | WP_008889355 |
| 11 | 13363-14328 | → | 321 | hypothetical protein | 27%  (90/333) | *Sphingobium ummariense* | WP_021316953 |
| 12 | 14479-15021 | ← | 180 | site-specific recombinase-resolvase | 815%  (146/180) | *P. marcusii* DSM 11574 (plasmid pMARC5) | YP_006965351 |
| 13 | 15018-15371 | ← | 117 | toxin of toxin-antitoxin system | 59%  (51/87) | *Rhodobacterales bacterium* Y4I | WP_008558483 |
| 14 | 15325-15567 | ← | 80 | antitoxin of toxin-antitoxin system | 68%  (53/78) | *Sinorhizobium fredii* USDA 257 | YP_006398227 |
| 15 | 15885-16049 | → | 54 | hypothetical protein | 43%  (22/51) | *Bradyrhizobium* sp. YR681 | WP_008129330 |
| 16 | 16323-16511 | ← | 62 | transposase, truncated | 94%  (59/62) | *S. fredii* USDA 257 | YP_006396430 |
| 17 | 16531-16737 | → | 68 | transposase, truncated | 92%  (57/62) | *Pelagibaca bermudensis* | WP_007801170 |
| 18 | 16802-17044 | ← | 80 | hypothetical protein | 43%  (28/65) | *P. denitrificans* PD1222 | YP_913895 |
| 19 | 17209-18612 | ← | 467 | mobilization protein A (MobA) | 60%  (289/485) | *P. aminophilus* JCM 7686 (plasmid pAMI7) | YP_004103195 |
| 20 | 19254-19712 | → | 152 | mobilization protein C (MobC) | 85%  (68/80) | *P. aminophilus* JCM 7686 (plasmid pAMI7) | YP_004103196 |
| 21 | 19709-20746 | → | 345 | TraG conjugal transfer transmembrane protein | 60%  (131/217) | *P. aminophilus* JCM 7686 (plasmid pAMI7) | YP_004103197 |
| **Plasmid pLM20P5 (28489 bp)** | | | | | | | |
| 1 | 1-843 | ← | 280 | replication initiator protein | 53 %  (142/270) | *P. marcusii* DSM 11574 (plasmid pMARC5) | YP_006965327 |
| 2 | 840-1523 | ← | 227 | resolvase | 69% (157/226) | *Rhodobacter sphaeroides* ATCC 17025 (plasmid pRSPA03) | YP_001170449 |
| 3 | 1679-2293 | → | 204 | partitioning protein ParA | 76%  (155/204) | *Loktanella hongkongensis* | WP_017930027 |
| 4 | 2286-2552 | → | 88 | hypothetical protein | 78%  (65/83) | *Rhodobacterales bacterium* Y4I | WP_008553871 |
| 5 | 2726-3421 | → | 231 | allantoate amidohydrolase | 56%  (66/117) | *Paracoccus* sp. TRP | WP_010396036 |
| 6 | 3418-4905 | → | 495 | microcystin LR degradation protein MlrC | 81%  (397/491) | *P. denitrificans* PD1222 | YP_918026 |
| 7 | 5064-6179 | ← | 371 | histidinol-phosphate aminotransferase | 47%  (167/353) | *Sinorhizobium meliloti* 1021 (plasmid pSymA) | NP_435452 |
| 8 | 6196-7581 | ← | 461 | D-lactate dehydrogenase | 57%  (256/452) | *Rhodospirillum rubrum* ATCC 11170 | YP_426387 |
| 9 | 7642-9201 | ← | 519 | ABC-type dipeptide transport system, periplasmic component | 46%  (235/515) | *Pseudaminobacter salicylatoxidans* | WP_019173648 |
| 10 | 9371-10300 | → | 322 | LysR family transcriptional regulator | 39%  (114/293) | *Burkholderia* sp. TJI49 | WP_009692145 |
| 11 | 10383-11423 | → | 346 | vanillate O-demethylase oxygenase, (2Fe-2S)-binding protein | 78%  (267/344) | *Oceanibulbus indolifex* | WP_007118608 |
| 12 | 11426-12598 | → | 390 | flavohemoprotein-like protein, oxidoreductase | 45%  (181/401 | *Moritella* sp. PE36 | WP_006031546 |
| 13 | 12949-13662 | ← | 237 | succinylglutamate desuccinylase/aspartoacylase family protein, ectoine utilization protein EutE | 92%  (92/210) | *Arthrobacter crystallopoietes* | WP_005269271 |
| 14 | 13659-15314 | ← | 551 | ABC transporter | 69%  (372/540) | *Labrenzia alexandrii* | WP_008192396 |
| 15 | 15319-16122 | ← | 267 | ABC-type dipeptide/oligopeptide/nickel transport system, permease component | 66%  (175/267) | *Rubellimicrobium thermophilum* | WP_021098983 |
| 16 | 16181-17137 | ← | 318 | ABC-type dipeptide/oligopeptide/nickel transport system, permease component | 64%  (205/318) | *Marinomonas* sp. MED121 | WP_009831915 |
| 17 | 17238-17978 | → | 246 | GntR family transcriptional regulator, histidine utilization repressor (HutR) | 57% (135/235) | *Roseobacter* sp. MED193 | WP_009807731 |
| 18 | 17999-19369 | ← | 456 | *N*-formimino-L-glutamate deiminase (HutF) | 69%  (308/445) | *Pannonibacter phragmitetus* | WP_019966387 |
| 19 | 19461-20654 | → | 397 | imidazolonepropionase (HutI) | 72%  (285/395) | *Pannonibacter phragmitetus* | WP_019966386 |
| 20 | 20648-22168 | → | 506 | histidine ammonia-lyase (HutH) | 77%  (385/501) | *Pannonibacter phragmitetus* | WP_019966385 |
| 21 | 22165-22968 | → | 267 | *N*-formylglutamate amidohydrolase (HutG) | 80%  (225/259) | *Paracoccus* sp. N5 | WP_017999503 |
| 22 | 23060-24742 | → | 560 | urocanate hydratase (HutU) | 89%  (499/560) | *Pannonibacter phragmitetus* | WP_019966383 |
| 23 | 24739-25386 | → | 215 | HutD-family protein | 47%  (82/174) | *Rhizobium leguminosarum* | WP_017957870 |
| 24 | 25335-25961 | ← | 208 | transposase, truncated | 76%  (116/153) | *Gluconobacter frateurii* NBRC 103465 | GAD09052 |
| 25 | 26004-26432 | → | 142 | hypothetical protein | 80%  (90/112) | *Novispirillum itersonii* | WP_019643580 |
| 26 | 26588-26842 | → | 84 | toxin of toxin-antitoxin system, HicA-like | 94%  (79/84) | *Rhodobacter sphaeroides* ATCC 17025 (plasmid pRSPA02) | YP_001170337 |
| 27 | 26839-27171 | → | 110 | antitoxin of toxin-antitoxin system, HicB-like | 98%  (108/110) | *Rhodobacter sphaeroides* ATCC 17025 (plasmid pRSPA02) | YP_001170338 |
| 28 | 27197-27982 | ← | 261 | hypothetical protein | 58%  (76/130) | *Arhodomonas aquaeolei* | WP_018718287 |
