# Supplementary material for: Diversity and role of plasmids in adaptation of bacteria inhabiting the Lubin copper mine in Poland, an environment rich in heavy metals
Source: Front Microbiol. 2015 Mar 3;6:152. doi: 10.3389/fmicb.2015.00152 (PMC4447125; doi:10.3389/fmicb.2015.00152)
Supplement: Supplementary file 9 [file Table4.DOC]

**Table S4.** Genes located within plasmid pLM16A1 of *Achromobacter* sp. LM16.

| Gene no. | **Coding region**  **(bp)** | **Strand** | **Protein size (aa)** | **Possible function** | **Best BLAST hits** | | |
| --- | --- | --- | --- | --- | --- | --- | --- |
|  |  |  |  |  | **% identity (aa)** | **Organism** | **GenBank accession no.** |
| **Plasmid pLM16A1 (25026 bp)** | | | | | | | |
| 1 | 716-1834 | ← | 372 | replication initiation protein | 100%  (372/372) | *Pseudomonas aeruginosa* | WP_003159118 |
| 2 | 1888-2112 | ← | 74 | partitioning protein B (ParB) | 100%  (74/74) | *Pseudomonas aeruginosa* | WP_003159117 |
| 3 | 2133-2765 | ← | 210 | partitioning protein A (ParA) | 100%  (210/210) | *Serratia marcescens* | BAB71950 |
| 4 | 2847-3140 | ← | 97 | hypothetical protein | 91%  (88/97) | *Pseudomonas aeruginosa* | WP_020750546 |
| 5 | 3137-3802 | ← | 221 | resolvase | 99%  (220/221) | *Pseudomonas aeruginosa* | WP_003159114 |
| 6 | 3979-4197 | → | 72 | hypothetical protein | 97%  (70/72) | *Acidovorax sp.* JS42 (plasmid pAOVO02) | YP_974086 |
| 7 | 4194-5150 | → | 318 | DNA-cytosine methyltransferase | 97%  (307/318) | *Acidovorax sp.* JS42 (plasmid pAOVO02) | YP_974087 |
| 8 | 5150-6013 | → | 287 | type II DNA restriction endonuclease | 100%  (277/277) | *Pseudomonas aeruginosa* COL-1 (plasmid pNOR-2000) | YP_007509598 |
| 9 | 6180-6386 | → | 68 | antitoxin of toxin-antitoxin system | 99%  (67/68) | *Pseudomonas aeruginosa* COL-1 (plasmid pNOR-2000) | YP_007509596 |
| 10 | 6449-6787 | ← | 112 | hypothetical protein | 100%  (112/112) | *Pseudomonas aeruginosa* C (plasmid pKLC102) | AAP22631 |
| 11 | 6883-7818 | ← | 311 | serine protease | 99%  (310/311) | *Pseudomonas aeruginosa* C (plasmid pKLC102) | AAP22630 |
| 12 | 7805-8023 | ← | 72 | hypothetical protein | 100%  (72/72) | *Pseudomonas aeruginosa* C (plasmid pKLC102) | AAP22629 |
| 13 | 7817-8389 | → | 190 | hypothetical protein | 100%  (190/190) | *Pseudomonas aeruginosa* C (plasmid pKLC102) | AAP22628 |
| 14 | 8571-12092 | ← | 1173 | conjugal transfer protein TraA | 99%  1167/1173 | *Pseudomonas aeruginosa* C (plasmid pKLC102) | AAP22627 |
| 15 | 12109-12366 | → | 85 | conjugal transfer protein TraC | 100%  (85/85) | *Pseudomonas aeruginosa* C (plasmid pKLC102) | AAP22626 |
| 16 | 12389-12610 | → | 73 | conjugal transfer protein TraD | 99%  (72/73) | *Pseudomonas aeruginosa* C (plasmid pKLC102) | AAP22625 |
| 17 | 12645-15167 | → | 840 | conjugal transfer protein TraG | 99%  (838/840) | *Pseudomonas aeruginosa* C (plasmid pKLC102) | AAP22624 |
| 18 | 15178-15570 | → | 130 | hypothetical protein | 94%  (122/130) | *Pseudomonas aeruginosa* C (plasmid pKLC102) | AAP22623 |
| 19 | 15742-16653 | ← | 303 | KfrA protein | 88%  (267/303) | *Pseudomonas aeruginosa* | WP_003159119 |
| 20 | 16825-19791 | ← | 988 | transposase | 100%  (988/988) | *Cupriavidus metallidurans* CH34 (plasmid pMOL30) | YP_145631 |
| 21 | 19794-20354 | ← | 186 | resolvase | 100%  (186/186) | *Pseudomonas aeruginosa* | WP_016254288 |
| 22 | 20485-21474 | ← | 329 | hypothetical protein, Urf2 protein | 100%  (329/329) | uncultured bacterium (plasmid pB10) | NP_858032 |
| 23 | 21471-21707 | ← | 78 | mercury resistance protein MerE | 100%  (78/78) | uncultured bacterium (plasmid pB10) | NP_858033 |
| 24 | 21704-22069 | ← | 121 | transcriptional regulator MerD | 100%  (121/121) | uncultured bacterium (plasmid pB10) | NP_858034 |
| 25 | 22087-23772 | ← | 561 | mercuric reductase MerA | 100%  (561/561) | *Shigella flexneri* 5a str. M90T (plasmid pWR501) | NP_085424 |
| 26 | 23844-24119 | ← | 91 | periplasmic mercury ion binding protein MerP | 100%  (91/91) | uncultured bacterium (plasmid pB10) | NP_858036 |
| 27 | 24132-24482 | ← | 116 | mercuric transport protein MerT | 100%  (116/116) | *Cupriavidus metallidurans* CH34 (plasmid pMOL30) | YP_145638 |
| 28 | 24554-24988 | → | 144 | transcriptional regulator MerR | 100%  (144/144) | uncultured bacterium (plasmid pB10) | NP_858038 |
