# Supplementary material for: Diversity and role of plasmids in adaptation of bacteria inhabiting the Lubin copper mine in Poland, an environment rich in heavy metals
Source: Front Microbiol. 2015 Mar 3;6:152. doi: 10.3389/fmicb.2015.00152 (PMC4447125; doi:10.3389/fmicb.2015.00152)
Supplement: Supplementary file 10 [file Table5.DOC]

**Table S5.** Genes and tRNA encoding sequences located within plasmid pLM21S1 of *Sinorhizobium* sp. LM21.

| Gene no. | **Coding region**  **(bp)** | **Strand** | **Protein size (aa)** | **Possible function** | **Best BLAST hits** | | |
| --- | --- | --- | --- | --- | --- | --- | --- |
|  |  |  |  |  | **% identity (aa)** | **Organism** | **GenBank accession no.** |
| **Plasmid pLM21S1 (117539 bp)** | | | | | | | |
| 1 | 1-1110 | ← | 369 | replication protein C (RepC) | 72%  (241/334) | *Rhizobium etli* CFN42 (phage RHEph10) | AGC36083 |
| 2 | 1825-2217 | → | 130 | hypothetical protein | 44%  (48/110) | *R. etli* CFN42 (phage RHEph10) | AGC36085 |
| 3 | 2339-2566 | → | 75 | hypothetical protein | 55%  (17/31) | *R. etli* CFN42 (phage RHEph10) | AGC36086 |
| 4 | 2614-5022 | → | 802 | cobalamin biosynthesis protein CobT | 38%  (333/875) | *R. etli* CFN42 (phage RHEph10) | AGC36087 |
| 5 | 5133-6449 | → | 438 | cobaltochelatase subunit CobS | 54%  (226/421) | *R. etli* CFN42 (phage RHEph10) | AGC36088 |
| 6 | 6449-7090 | → | 213 | RNA polymerase sigma factor protein | 36%  (72/198) | *R. etli* CFN42 (phage RHEph10) | AGC36089 |
| 7 | 7098-7646 | → | 182 | hypothetical protein | 47%  (84/179) | *R. etli* CFN42 (phage RHEph10) | AGC36090 |
| 8 | 7652-11020 | → | 1122 | DNA polymerase III subunit alpha | 64%  (731/1146) | *R. etli* CFN42 (phage RHEph10) | AGC36092 |
| 9 | 11158-11574 | → | 138 | hypothetical protein | no similarity found | | |
| 10 | 11856-12386 | → | 176 | hypothetical protein | 54%  (89/164) | *R. etli* CFN42 (phage RHEph10) | AGC36093 |
| 11 | 12464-12727 | → | 87 | hypothetical protein | no similarity found | | |
| 12 | 12853-13923 | → | 356 | hypothetical protein | 46%  (121/262) | *R. etli* CFN42 (phage RHEph10) | AGC36095 |
| 13 | 14039-14989 | → | 316 | 5'-3' exonuclease | 50%  (161/320) | *R. etli* CFN42 (phage RHEph10) | AGC36096 |
| 14 | 15009-16172 | → | 387 | RecA-like recombinase | 47%  (168/359) | *R. etli* CFN42 (phage RHEph10) | AGC36099 |
| 15 | 16172-16417 | → | 81 | hypothetical protein | no similarity found | | |
| 16 | 16892- 17356 | → | 154 | hypothetical protein | 36%  (45/124) | *Phaeospirillum molischianum* | WP_002731428 |
| 17 | 17440-18534 | → | 364 | DNA repair exonuclease protein, subunit 1 | 51%  (186/366) | *R. etli* CFN42 (phage RHEph10) | AGC36103 |
| 18 | 18593-18934 | → | 113 | hypothetical protein | 32%  (38/117) | *R. etli* CFN42 (phage RHEph10) | AGC36104 |
| 19 | 18931-19173 | → | 80 | head-tail adaptor protein | 31%  (32/102) | *Sinorhizobium meliloti* | WP_017270686 |
| 20 | 19170-21107 | → | 645 | DNA repair exonuclease protein, subunit 2 | 46%  (307/667) | *R. etli* CFN42 (phage RHEph10) | AGC36107 |
| 21 | 21104-21301 | → | 65 | hypothetical protein | no similarity found | | |
| 22 | 21301-21501 | → | 66 | hypothetical protein | no similarity found | | |
| 23 | 21498-21770 | → | 90 | hypothetical protein | no similarity found | | |
| 24 | 21834-22304 | → | 156 | hypothetical protein | 33%  (41/124) | *R. etli* CFN42 (phage RHEph10) | AGC36112 |
| 25 | 22304-22798 | → | 164 | HNH endonuclease | 47%  (75/161) | *Enterobacter cloacae* subsp. cloacae NCTC 9394 | YP_007846311 |
| 26 | 22795-23367 | → | 190 | hypothetical protein | 53%  (100/188) | *R. etli* CFN42 (phage RHEph10) | AGC36113 |
| 27 | 23436-24440 | → | 334 | ribonucleoside-diphosphate reductase protein, beta subunit | 73%  (233/320) | *R. etli* CFN42 (phage RHEph10) | AGC36114 |
| 28 | 24456-26138 | → | 560 | ribonucleoside-diphosphate reductase protein, alpha subunit | 76%  (425/560) | *R. etli* CFN42 (phage RHEph10) | AGC36115 |
| 29 | 26157- 26360 | → | 67 | glutaredoxin | 48%  (31/65) | *R. etli* CFN42 (phage RHEph10) | AGC36116 |
| 30 | 26454-26912 | → | 152 | hypothetical protein | no similarity found | | |
| 31 | 26917-27918 | → | 333 | thymidylate synthase | 48%  (159/330) | *Sulfitobacter* sp. EE-36 (phage EE36phi1) | YP_002898958 |
| 32 | 27915-28172 | → | 85 | hypothetical protein | 45%  (37/82) | *R. etli* CFN42 (phage RHEph10) | AGC36121 |
| 33 | 28165- 28419 | → | 84 | hypothetical protein | no similarity found | | |
| 34 | 28428-28619 | → | 63 | hypothetical protein | no similarity found | | |
| 35 | 28644-29093 | → | 149 | deoxyuridine 5'-triphosphate nucleotidohydrolase | 59%  (85/144) | *Nautilia profundicola* AmH | YP_002606781 |
| 36 | 29095-29715 | → | 206 | hypothetical protein | 39%  (79/203) | *R. etli* CFN42 (phage RHEph10) | AGC36126 |
| 37 | 29912-30361 | → | 149 | hypothetical protein | no similarity found | | |
| 38 | 30832-31023 | → | 63 | hypothetical protein | 59%  (44/59) | *Sphingobium sp.* HDIP04 | WP_021223420 |
| 39 | 31980-32480 | → | 166 | hypothetical protein | 53%  (32/60) | *Halosarcina pallida* | WP_008386369 |
| 40 | 32504-32579 | → | - | tRNA-Cys(GCA) | - | *-* | - |
| 41 | 32669-35077 | → | 802 | rIIA-like protein | 36%  (300/832) | *R. etli* CFN42 (phage RHEph10) | AGC36127 |
| 42 | 35074-36666 | → | 530 | rIIB-like protein | 43%  (234/541) | *R. etli* CFN42 (phage RHEph10) | AGC36128 |
| 43 | 36758-37369 | → | 203 | hypothetical protein | 48%  (91/190) | *Ralstonia pickettii* 12J | YP_001899889 |
| 44 | 37379-38062 | → | 227 | hypothetical protein | 71%  (140/197) | *R. etli* CFN42 (phage RHEph10) | AGC36129 |
| 45 | 38096-38347 | → | 83 | hypothetical protein | 35%  (24/68) | *R. etli* CFN42 (phage RHEph10) | AGC36131 |
| 46 | 38379-38603 | → | 76 | hypothetical protein | no similarity found | | |
| 47 | 38600-39145 | → | 181 | HNH endonuclease family protein | 53%  (102/194) | *R. etli* CFN42 (phage RHEph10) | AGC36132 |
| 48 | 39267-39836 | → | 189 | DNA polymerase III subunit epsilon | 52%  (99/190) | *R. etli* CFN42 (phage RHEph10) | AGC36135 |
| 49 | 39826-40365 | → | 179 | HNH endonuclease | 44%  (72/165) | Deep-sea thermophilic phage D6E | YP_007010936 |
| 50 | 40445-41221 | → | 258 | hypothetical protein | 45%  (95/210) | *R. etli* CFN42 (phage RHEph10) | AGC36136 |
| 51 | 41347-41628 | → | 93 | hypothetical protein | no similarity found | | |
| 52 | 41639-42073 | → | 144 | hypothetical protein | no similarity found | | |
| 53 | 42234-42596 | → | 120 | hypothetical protein | 68%  (78/114) | *Rhizobium freirei* | WP_004110174 |
| 54 | 42703-43104 | → | 133 | hypothetical protein | 65%  (74/114) | EBPR siphovirus 2 | AEI71071 |
| 55 | 43116-43526 | → | 136 | hypothetical protein | 48%  (63/131) | *Salinispora pacifica* | WP_018810208 |
| 56 | 43617-43808 | → | 63 | hypothetical protein | no similarity found | | |
| 57 | 43808-44845 | → | 345 | bifunctional nicotinamide mononucleotide adenylyltransferase/ADP-ribose pyrophosphatase (NadM) | 48%  (166/348) | *Labrenzia* sp. C1B10 | WP_023003877 |
| 58 | 44847-46292 | → | 481 | nicotinate phosphoribosyltransferase (NadV) | 61%  (274/449) | *Erythrobacter* sp. SD-21 | WP_006832942 |
| 59 | 46628-47482 | → | 284 | SPFH domain protein | 42%  118/281 | *Pseudomonas* sp. M1 | WP_009620067 |
| 60 | 47562-47912 | → | 116 | hypothetical protein | no similarity found | | |
| 61 | 47997-48410 | → | 137 | hypothetical protein | no similarity found | | |
| 62 | 49271-49477 | ← | 68 | hypothetical protein | no similarity found | | |
| 63 | 49757-50161 | → | 134 | hypothetical protein | 29%  (33/112) | *Rhodobacter sphaeroides* ATCC 17025 (plasmid pRSPA02) | YP_001170374 |
| 64 | 50183-50419 | → | 78 | hypothetical protein | no similarity found | | |
| 65 | 50687-51001 | → | 104 | hypothetical protein | 28%  (29/104) | *R. etli* CFN42 (phage RHEph10) | AGC36145 |
| 66 | 51003-51260 | → | 85 | hypothetical protein | 43%  (23/54) | *R. etli* CFN42 (phage RHEph10) | AGC36147 |
| 67 | 51319-51624 | → | 101 | hypothetical protein | 37%  (33/89) | *Rhodanobacter fulvus* | WP_007079967 |
| 68 | 51621-51857 | → | 78 | hypothetical protein | no similarity found | | |
| 69 | 51854-52522 | → | 222 | hypothetical protein | no similarity found | | |
| 70 | 52522-52869 | → | 115 | hypothetical protein | 43%  (49/115) | *R. etli* CFN42 (phage RHEph10) | AGC36153 |
| 71 | 52990-53271 | → | 93 | hypothetical protein | no similarity found | | |
| 72 | 53276-53530 | → | 84 | hypothetical protein | no similarity found | | |
| 73 | 53574-54128 | → | 184 | metal dependent phosphohydrolase (SpoT) | 51%  (88/172) | *R. etli* CFN42 (phage RHEph10) | AGC36149 |
| 74 | 54125-54676 | → | 183 | hypothetical protein | no similarity found | | |
| 75 | 54785-55261 | → | 158 | hypothetical protein | no similarity found | | |
| 76 | 55261-55671 | → | 136 | hypothetical protein | 41%  (58/141) | *R. etli* CFN42 (phage RHEph10) | AGC36165 |
| 77 | 55656-55874 | → | 72 | hypothetical protein | 73%  (45/62) | *R. etli* CFN42 (phage RHEph10) | AGC36169 |
| 78 | 55871-56197 | → | 108 | hypothetical protein | no similarity found | | |
| 79 | 56185-56394 | → | 69 | hypothetical protein | no similarity found | | |
| 80 | 56630-56920 | → | 96 | hypothetical protein | 55%  (55/100) | *Pseudomonas aeruginosa* | WP_015648597 |
| 81 | 57422-57757 | → | 111 | hypothetical protein | no similarity found | | |
| 82 | 58127-58429 | → | 100 | hypothetical protein | 47%  (45/96) | *R. etli* CFN42 (phage RHEph10) | AGC36163 |
| 83 | 58529-59026 | → | 165 | hypothetical protein | no similarity found | | |
| 84 | 59104-59559 | → | 151 | hypothetical protein | 31%  (30/96) | *R. etli* CFN42 (phage RHEph10) | AGC36175 |
| 85 | 59559-59840 | → | 93 | hypothetical protein | no similarity found | | |
| 86 | 59837-60220 | → | 127 | hypothetical protein | no similarity found | | |
| 87 | 60381-60617 | → | 78 | hypothetical protein | no similarity found | | |
| 88 | 60614-60823 | → | 69 | hypothetical protein | no similarity found | | |
| 89 | 60946-61326 | → | 126 | hypothetical protein | 47%  (56/118) | *Loktanella* phage pCB2051-A | YP_007674971 |
| 90 | 61323-61532 | → | 69 | hypothetical protein | 57%  (17/30) | *Ahrensia* sp. R2A130 | WP_009465056 |
| 91 | 61511-61741 | → | 76 | hypothetical protein | no similarity found | | |
| 92 | 61738-62001 | → | 87 | hypothetical protein | no similarity found | | |
| 93 | 62134-62403 | → | 89 | hypothetical protein | no similarity found | | |
| 94 | 63793-64770 | → | 325 | hypothetical protein | no similarity found | | |
| 95 | 64976-65467 | → | 163 | hypothetical protein | no similarity found | | |
| 96 | 65812-66189 | → | 125 | hypothetical protein | no similarity found | | |
| 97 | 67138-67911 | ← | 257 | hypothetical protein | no similarity found | | |
| 98 | 68117-68193 | → | - | tRNA-Met(CAT) | - | - | - |
| 99 | 68198-68273 | → | - | tRNA-Asn(GTT) | - | - | - |
| 100 | 68295-68732 | → | 145 | hypothetical protein | 41%  (30/73) | *R. etli* CFN42 (phage RHEph10) | AGC36194 |
| 101 | 68729-69352 | → | 207 | ATP-dependent protease | 53%  (78/148) | *R. etli* CFN42 (phage RHEph10) | AGC36195 |
| 102 | 69377-69583 | → | 68 | hypothetical protein | no similarity found | | |
| 103 | 69901-69644 | ← | 85 | putative ParB protein | 38%  (28/73) | *R. etli* CFN42 (phage RHEph10) | AGC36197 |
| 104 | 69898-70545 | ← | 215 | partition protein parA | 52%  (111/213) | *R. etli* CFN42 (phage RHEph10) | AGC36198 |
| 105 | 70636-71067 | ← | 143 | hypothetical protein | no similarity found | | |
| 106 | 71067-71621 | ← | 184 | hypothetical protein | 81%  (146/180) | *R. etli* CFN42 (phage RHEph10) | AGC36200 |
| 107 | 71929-73497 | ← | 522 | DEAD/DEAH box helicase | 60%  (389/532) | *R. etli* CFN42 (phage RHEph10) | AGC36202 |
| 108 | 73576-74325 | → | 249 | chromosome partitioning protein ParB | 53%  (123/234) | *R. etli* CFN42 (phage RHEph10) | AGC36203 |
| 109 | 74325-74987 | → | 220 | chromosome partitioning protein ParB | 57%  (121/213) | *R. etli* CFN42 (phage RHEph10) | AGC36205 |
| 110 | 75013-75453 | → | 146 | hypothetical protein | 40%  (53/132) | *R. etli* CFN42 (phage RHEph10) | AGC36206 |
| 111 | 75446-76084 | → | 212 | ABC transporter | 66%  (140/212) | *R. etli* CFN42 (phage RHEph10) | AGC36207 |
| 112 | 76081-76377 | → | 98 | hypothetical protein | 37%  (37/100) | *R. etli* CFN42 (phage RHEph10) | AGC36208 |
| 113 | 76370-76561 | → | 63 | hypothetical protein | 73%  (40/55) | *R. etli* CFN42 (phage RHEph10) | AGC36209 |
| 114 | 76554-76877 | → | 107 | hypothetical protein | 55%  (59/108) | *Ralstonia* phage RSL1 | YP_001950135 |
| 115 | 76986-77855 | → | 289 | hypothetical protein | 65%  (184/285) | *R. etli* CFN42 (phage RHEph10) | AGC36210 |
| 116 | 77855-78106 | → | 83 | hypothetical protein | 41%  (31/76) | *R. etli* CFN42 (phage RHEph10) | AGC36211 |
| 117 | 78255-78908 | → | 217 | hypothetical protein | 45%  (100/221) | *R. etli* CFN42 (phage RHEph10) | AGC36212 |
| 118 | 78908-80176 | → | 422 | terminase protein, large subunit | 66%  (278/424) | *R. etli* CFN42 (phage RHEph10) | AGC36213 |
| 119 | 80235-81866 | → | 543 | phage portal protein | 59%  (312/530) | *R. etli* CFN42 (phage RHEph10) | AGC36214 |
| 120 | 81962-82912 | → | 316 | hypothetical protein | 40%  (100/249) | *Acinetobacter baumannii* MDR-TJ (plasmid pABTJ2) | YP_007569982 |
| 121 | 83061-83960 | → | 299 | phage capsid protein | 48%  (142/297) | *Acinetobacter baumannii* | WP_001130337 |
| 122 | 84035-84721 | → | 228 | hypothetical protein | 38%  (85/222) | *R. etli* CFN42 (phage RHEph10) | AGC36048 |
| 123 | 84725-85564 | → | 279 | hypothetical protein | 44%  (123/279) | *R. etli* CFN42 (phage RHEph10) | AGC36049 |
| 124 | 85561-86316 | → | 251 | minor capsid protein | 35%  (91/257) | *R. etli* CFN42 (phage RHEph10) | AGC36050 |
| 125 | 86303-86650 | → | 115 | hypothetical protein | 32%  (35/111) | *R. etli* CFN42 (phage RHEph10) | AGC36051 |
| 126 | 86647-87141 | → | 164 | head-to-tail joining protein | 38%  (59/154) | *R. etli* CFN42 (phage RHEph10) | AGC36052 |
| 127 | 87138-87554 | → | 138 | hypothetical protein | 42%  (56/134) | *R. etli* CFN42 (phage RHEph10) | AGC36053 |
| 128 | 87681-88511 | → | 276 | tail protein | 64%  (176/277) | *R. etli* CFN42 (phage RHEph10) | AGC36054 |
| 129 | 88599-88955 | → | 118 | hypothetical protein | 35%  (26/74) | *R. etli* CFN42 (phage RHEph10) | AGC36055 |
| 130 | 88973-89305 | → | 110 | hypothetical protein | 39%  (33/85) | *R. etli* CFN42 (phage RHEph10) | AGC36056 |
| 131 | 89339-93979 | → | 1546 | phage tail tape measure protein | 38%  (615/1618) | *R. etli* CFN42 (phage RHEph10) | AGC36057 |
| 132 | 93976-94329 | → | 117 | phage minor tail protein | 49%  (57/117) | *R. etli* CFN42 (phage RHEph10) | AGC36058 |
| 133 | 94400-95110 | → | 236 | phage minor tail protein | 60%  (141/235) | *R. etli* CFN42 (phage RHEph10) | AGC36059 |
| 134 | 95107-95898 | → | 263 | phage tail assembly protein | 49%  (123/252) | *R. etli* CFN42 (phage RHEph10) | AGC36060 |
| 135 | 95891-96544 | → | 217 | phage tail assembly protein | 56%  (113/201) | *R. etli* CFN42 (phage RHEph10) | AGC36061 |
| 136 | 96541-101679 | → | 1712 | phage tail fiber protein | 49%  (609/1255) | *R. etli* CFN42 (phage RHEph10) | AGC36062 |
| 137 | 101682-102101 | → | 139 | hypothetical protein | 41%  (32/78) | *R. etli* CFN42 (phage RHEph10) | AGC36066 |
| 138 | 102098-102508 | → | 136 | hypothetical protein | 46%  (51/111) | *R. etli* CFN42 (phage RHEph10) | AGC36067 |
| 139 | 102584-102931 | → | 115 | phage tape measure protein | 48%  (53/110) | *R. etli* CFN42 (phage RHEph10) | AGC36068 |
| 140 | 102931-103545 | → | 204 | phage related lysozyme protein | 83%  (169/204) | *R. etli* CFN42 (phage RHEph10) | AGC36070 |
| 141 | 103545-103889 | → | 114 | hypothetical protein | 52%  (59/114) | *R. etli* CFN42 (phage RHEph10) | AGC36071 |
| 142 | 103945-104109 | → | 54 | hypothetical protein | 40%  (17/43) | *R. etli* CFN42 (phage RHEph10) | AGC36072 |
| 143 | 104184-106025 | → | 613 | hypothetical protein | 67%  (407/609) | *R. etli* CFN42 (phage RHEph10) | AGC36073 |
| 144 | 106094-106444 | → | 116 | hypothetical protein | 42%  (49/116) | *Roseobacter* sp. MED193 | WP_009811268 |
| 145 | 106444-109929 | → | 1161 | hypothetical protein | no similarity found | | |
| 146 | 109987-110379 | → | 130 | hypothetical protein | 37%  (37/100) | *R. etli* Brasil5 (phage RHEph01) | AGC35559 |
| 147 | 110477-111244 | → | 255 | phosphate starvation-inducible protein phoH | 54%  (151/279) | *R. etli* CFN42 (phage RHEph10) | AGC36074 |
| 148 | 111341-112093 | → | 250 | hypothetical protein | 44%  (104/238) | *R. etli* CFN42 (phage RHEph10) | AGC36075 |
| 149 | 112170-113501 | → | 443 | DNA helicase DnaB-like protein | 59%  (255/434) | *R. etli* CFN42 (phage RHEph10) | AGC36076 |
| 150 | 113504-114622 | → | 372 | DNA primase | 63%  (236/375) | *R. etli* CFN42 (phage RHEph10) | AGC36077 |
| 151 | 114767-115291 | → | 174 | hypothetical protein | 27%  (49/180) | *R. etli* CFN42 (phage RHEph10) | AGC36078 |
| 152 | 115291-115863 | → | 190 | hypothetical protein | 39%  (75/193) | *R. etli* CFN42 (phage RHEph10) | AGC36080 |
| 153 | 115878-117404 | → | 508 | ATP-dependent DNA ligase | 42%  (218/513) | *R. etli* CFN42 (phage RHEph10) | AGC36081 |
