# Supplementary material for: Diversity and role of plasmids in adaptation of bacteria inhabiting the Lubin copper mine in Poland, an environment rich in heavy metals
Source: Front Microbiol. 2015 Mar 3;6:152. doi: 10.3389/fmicb.2015.00152 (PMC4447125; doi:10.3389/fmicb.2015.00152)
Supplement: Supplementary file 11 [file Table6.DOC]

**Table S6.** Genes located within plasmids pLM19O1 and pLM19O2 of *Ochrobactrum* sp. LM19.

| Gene no. | **Coding region**  **(bp)** | **Strand** | **Protein size (aa)** | **Possible function** | **Best BLAST hits** | | |
| --- | --- | --- | --- | --- | --- | --- | --- |
|  |  |  |  |  | **% identity (aa)** | **Organism** | **GenBank accession no.** |
| **Plasmid pLM19O1 (78679 bp)** | | | | | | | |
| 1 | 1-1224 | ← | 407 | replication protein C (RepC) | 90%  (368/407) | *Ochrobactrum anthropi* ATCC 49188 (plasmid pOANT03) | YP_001373272 |
| 2 | 1380-2423 | ← | 347 | plasmid partitioning protein B (RepB) | 64%  (224/351) | *O. anthropi* ATCC 49188 (plasmid pOANT03) | YP_001373271 |
| 3 | 2427-3620 | ← | 397 | plasmid partitioning protein A (RepA) | 91%  (363/397) | *O. anthropi* ATCC 49188 (plasmid pOANT03) | YP_001373270 |
| 4 | 4077-5009 | → | 310 | hypothetical protein | 84%  (184/218) | *Agrobacterium tumefaciens* | WP_003501836 |
| 5 | 5198-5614 | → | 138 | major facilitator superfamily protein | 64%  (87/137) | *Rhizobium leguminosarum* bv. trifolii WSM2304 | YP_002281089 |
| 6 | 5889-6068 | → | 59 | hypothetical protein | 79%  (45/57) | *Rhizobium giardinii* | WP_018329053 |
| 7 | 6083-6982 | → | 229 | orotidine 5-phosphate decarboxylase | 74%  (222/302) | *Rhizobium giardinii* | WP_018324686 |
| 8 | 7140-8285 | ← | 381 | phage integrase family protein | 92%  (338/369) | *O. anthropi* ATCC 49188 (plasmid pOANT01) | YP_001373040 |
| 9 | 8275-8694 | ← | 139 | toxin of toxin-antitoxin | 91%  (127/139) | Ochrobactrum sp. EGD-AQ16 | WP_021585834 |
| 10 | 8694-8972 | ← | 92 | antitoxin of toxin-antitoxin | 97%  (77/79) | Ochrobactrum sp. EGD-AQ16 | WP_021585833 |
| 11 | 9135-10286 | → | 383 | hypothetical protein | 86%  (328/383) | *O. anthropi* ATCC 49188 (plasmid pOANT03) | YP_001373316 |
| 12 | 10466-10678 | ← | 70 | ATP-dependent DNA helicase | 71%  (47/66) | *Pseudomonas extremaustralis* | WP_010567759 |
| 13 | 10805-11557 | ← | 250 | hypothetical protein | 52%  (119/227) | *Bradyrhizobium* sp. ORS 285 | WP_006609592 |
| 14 | 11557-12171 | ← | 204 | hypothetical protein | 43%  (88/205) | *Bradyrhizobium* sp. ORS 285 | WP_006609593 |
| 15 | 12557-12748 | → | 63 | hypothetical protein | 76%  (29/38) | Ochrobactrum sp. EGD-AQ16 | WP_021586481 |
| 16 | 13268-14059 | → | 263 | hypothetical protein | no similarity found | | |
| 17 | 14059-15012 | → | 317 | hypothetical protein | 34%  (101/294) | *Microvirga* sp. WSM3557 | WP_009493551 |
| 18 | 15148-15534 | → | 128 | transposase | 93%  (109/117) | *Rhizobium leguminosarum* | WP_020046939 |
| 19 | 15531-15884 | → | 117 | transposase | 97%  (114/117) | *Agrobacterium radiobacter* K84 | YP_002546370 |
| 20 | 15953-17482 | → | 509 | transposase | 90%  (458/507) | *Rhizobium* sp. AP16 | WP_007691087 |
| 21 | 17515-17886 | → | 123 | hypothetical protein | 72%  (80/111) | *Pseudochrobactrum* sp. AO18b | WP_022709124 |
| 22 | 18094-18234 | → | 46 | hypothetical protein | no similarity found | | |
| 23 | 18370-20763 | ← | 797 | hypothetical protein | 55%  (424/770) | *Cupriavidus* sp. UYPR2.512 | WP_018309881 |
| 24 | 20923-21372 | ← | 149 | hypothetical protein | 52%  (76/147) | *Methylosinus trichosporium* | WP_003611816 |
| 25 | 21387-21848 | ← | 153 | UBA/THIF-type NAD/FAD binding protein | 59%  (64/109) | *M. trichosporium* | WP_003611812 |
| 26 | 22060-22347 | ← | 95 | cold shock protein (CspA) | 90%  (60/67) | *Ochrobactrum* sp. CDB2 | WP_007881014 |
| 27 | 23118-23375 | → | 85 | XRE family transcriptional regulator | 81%  (65/80) | *Ochrobactrum* sp. CDB2 | WP_007880799 |
| 28 | 23773-24498 | ← | 241 | hypothetical protein | no similarity found | | |
| 29 | 24759-25595 | → | 278 | hypothetical protein | no similarity found | | |
| 30 | 25777-26169 | ← | 130 | 5'-nucleotidase | 51%  (66/130) | *Rhodopseudomonas palustris* CGA009 | NP_948168 |
| 31 | 26194-26517 | ← | 107 | hypothetical protein | 41%  (41/100) | *Ochrobactrum* sp. CDB2 | WP_007881302 |
| 32 | 27051-27470 | → | 139 | SH3 type 3 domain-containing protein | 49%  (67/138) | *Hoeflea* sp. 108 | WP_018428964 |
| 33 | 27627-28013 | → | 128 | hypothetical protein | 49%  (60/122) | *Rhizobium* sp. 42MFCr.1 | WP_018859145 |
| 34 | 28325-29029 | → | 234 | transposase | 93%  (217/234) | *Ochrobactrum* sp. EGD-AQ16 | WP_021586543 |
| 35 | 29534-33211 | ← | 1225 | outer membrane autotransporter barrel domain-containing protein | 48%  (587/1216) | *Ochrobactrum* sp. CDB2 | WP_007878218 |
| 36 | 33413-33958 | ← | 181 | invasion-associated locus B (IalB) | 47%  (73/154) | *Chelatococcus* sp. GW1 | WP_019401774 |
| 37 | 34363-34497 | ← | 44 | hypothetical protein | no similarity found | | |
| 38 | 34668-35126 | → | 152 | hypothetical protein | 35%  (45/130) | *Oceaniovalibus guishaninsula* | WP_007427084 |
| 39 | 35160-36089 | ← | 309 | response regulator receiver protein | 72%  (218/301) | *Ochrobactrum* sp. CDB2 | WP_007878217 |
| 40 | 36113-38167 | ← | 684 | sensor signal transduction histidine kinase | 72%  (495/688) | *Ochrobactrum* sp. CDB2 | WP_007878216 |
| 41 | 39655-40029 | → | 124 | hypothetical protein | 42%  (54/129) | *Rhodopseudomonas palustris* CGA009 | NP_948168 |
| 42 | 40053-40313 | → | 86 | hypothetical protein | 62%  (53/86) | *Ochrobactrum* sp. EGD-AQ16 | WP_021587219 |
| 43 | 40339-41241 | → | 300 | hypothetical protein | 62%  (188/301) | *O. anthropi* | WP_010657925 |
| 44 | 41507-44251 | → | 914 | outer membrane autotransporter barrel domain-containing protein | 87%  (791/914) | *O. anthropi* | WP_010657928 |
| 45 | 44448-44726 | → | 92 | transposase | 98%  (90/92) | *Pseudochrobactrum* sp. AO18b | WP_022709784 |
| 46 | 44777-45352 | → | 191 | transposase | 87%  (165/189) | *Ochrobactrum intermedium* | WP_022569528 |
| 47 | 45530-46792 | ← | 420 | transposase | 100%  (420/420) | *Ochrobactrum anthropi* ATCC 49188 (plasmid pOANT01) | YP_001373139 |
| 48 | 47304-47573 | ← | 89 | hypothetical protein | no similarity found | | |
| 49 | 48048-48197 | → | 49 | transposase | 92%  (45/49) | *O. intermedium* | WP_006469792 |
| 50 | 48210-48731 | → | 173 | transposase | 84%  (147/174) | *O. anthropi* | WP_010660327 |
| 51 | 48833-49156 | → | 107 | transposase | 89%  (51/57) | *O. anthropi* ATCC 49188 (plasmid | YP_001373139 |
| 52 | 49131-49412 | ← | 93 | transposase | 95%  (88/93) | *Agrobacterium* sp. ATCC 31749 | WP_006311555 |
| 53 | 49509-49760 | ← | 83 | transposase | 90%  (39/51) | *Agrobacterium tumefaciens* Bo542 (plasmid pTiBo542) | YP_001967416 |
| 54 | 50341-53610 | → | 1089 | outer membrane autotransporter barrel domain-containing protein | 66%  (326/495) | *O. anthropi* ATCC 49188 (plasmid pOANT01) | YP_001373023 |
| 55 | 53680-53883 | → | 67 | hypothetical protein | no similarity found | | |
| 56 | 54059-54430 | → | 123 | transposase | 100%  (123/123) | *O. anthropi* ATCC 49188 (plasmid pOANT01) | YP_001373006 |
| 57 | 54495-55013 | → | 172 | transposase | 98%  (169/172) | *O. anthropi* ATCC 49188 (plasmid pOANT01) | YP_001373005 |
| 58 | 55064-55375 | ← | 103 | hypothetical protein | 61%  (51/84) | *Ochrobactrum* sp. CDB2 | WP_007878476 |
| 59 | 55701-56456 | ← | 251 | ErfK/YbiS/YcfS/YnhG family protein | 47%  (98/208) | *Shinella zoogloeoides* DD12 | ESS85568 |
| 60 | 56489-59497 | ← | 1002 | outer membrane autotransporter barrel domain-containing protein | 72%  (488/677) | *Agrobacterium tumefaciens* | WP_003523962 |
| 61 | 59871-60677 | → | 268 | transposase | 86%  (130/151) | *Agrobacterium radiobacter* K84 | YP_002542477 |
| 62 | 60701-61093 | ← | 130 | hypothetical protein | 93%  (84/90) | *Agrobacterium vitis* S4 | YP_002540135 |
| 63 | 61231-62760 | → | 509 | reverse transcriptase | 100%  (509/509) | *O. anthropi* | WP_010661544 |
| 64 | 63832-67509 | ← | 1225 | outer membrane autotransporter barrel domain-containing protein | 49%  (591/1216) | *Ochrobactrum* sp. CDB2 | WP_007878218 |
| 65 | 67711-68256 | ← | 181 | invasion-associated locus B (IalB) | 47%  (73/154) | *Chelatococcus* sp. GW1 | WP_019401774 |
| 66 | 68808-69821 | → | 337 | transposase | 82%  (276/337) | *Sphingobium ummariense* | WP_021319705 |
| 67 | 70331-70489 | → | 52 | transposase | 96%  (50/52) | *Sinorhizobium medicae* | WP_018009754 |
| 68 | 70569-70961 | ← | 130 | transposase | 57%  (60/105) | *Rhizobium grahamii* | WP_016557759 |
| 69 | 71089-71316 | ← | 75 | transposase | 65%  (24/37) | *Rhodopseudomonas palustris* TIE-1 | YP_001990074 |
| 70 | 73228-74757 | ← | 509 | reverse transcriptase | 100%  (509/509) | *O. anthropi* | WP_010661544 |
| 71 | 74895-75287 | → | 130 | hypothetical protein | 93%  (84/90) | *Agrobacterium vitis* S4 | YP_002540135 |
| 72 | 75311-75763 | ← | 150 | transposase | 100%  (124/124) | *O. anthropi* | WP_010661543 |
| 73 | 75848-77209 | ← | 453 | transposase | 85%  (382/448) | *Agrobacterium radiobacter* K84 | YP_002542476 |
| 74 | 77405-77677 | ← | 90 | transposase | 99%  (88/89) | *O. anthropi* | WP_010661541 |
| 75 | 78153-78578 | ← | 141 | hypothetical protein | 91%  (129/141) | *O. anthropi* ATCC 49188 (plasmid pOANT03) | YP_001373273 |
| **Plasmid pLM19O2 (107804 bp)** | | | | | | | |
| 1 | 1-1215 | ← | 404 | replication protein C (RepC) | 72%  (284/395) | *Sinorhizobium fredii* HH103 | YP_005190736 |
| 2 | 1430-2419 | ← | 329 | plasmid partitioning protein B (RepB) | 48%  (158/329) | *Sinorhizobium meliloti* AK83 (plasmid pSINME01) | YP_004557969 |
| 3 | 2416-3633 | ← | 405 | plasmid partitioning protein A (RepA) | 73%  (282/388) | *S. meliloti* AK83 (plasmid pSINME01) | YP_004557970 |
| 4 | 3765-4580 | ← | 271 | type I restriction-modification system methyltransferase subunit | 64%  (144/225) | *Shinella zoogloeoides* DD12 | ESS77731 |
| 5 | 5205-6842 | ← | 545 | ParB-like partitioning protein | 55%  (313/567) | *Shinella zoogloeoides* DD12 | ESS77731 |
| 6 | 6953-7435 | ← | 160 | hypothetical protein | 82%  (131/160) | *Bradyrhizobium* sp. BTAi1 (plasmid pBBta01) | YP_001220578 |
| 7 | 7425-9299 | ← | 624 | integrase | 86%  (534/622) | *Bradyrhizobium* sp. BTAi1 (plasmid pBBta01) | YP_001220577 |
| 8 | 9303-11114 | ← | 603 | hypothetical protein | 84%  (504/603) | *Bradyrhizobium* sp. BTAi1 (plasmid pBBta01) | YP_001220576 |
| 9 | 11104-11997 | ← | 297 | transposase | 94%  (243/259) | *Bradyrhizobium* sp. BTAi1 (plasmid pBBta01) | YP_001220575 |
| 10 | 12430-12948 | ← | 172 | ParB-like partitioning protein | 71%  (72/102) | *Sinorhizobium meliloti* | WP_017265663 |
| 11 | 13891-14124 | → | 77 | hypothetical protein | no similarity found | | |
| 12 | 15159-15479 | ← | 106 | hypothetical protein | 75%  (80/106) | *Shinella zoogloeoides* DD12 | ESS77656 |
| 13 | 16219-16809 | ← | 196 | isochorismatase | 74%  (145/196) | *Streptomyces* sp. AA4 | WP_009083094 |
| 14 | 17210-18091 | ← | 293 | transposase | 95%  (277/293) | *Brucella ovis* ATCC 25840 | YP_001257978 |
| 15 | 18792-19061 | → | 89 | hypothetical protein | no similarity found | | |
| 16 | 19452-19772 | ← | 106 | hypothetical protein | 75%  (80/106) | *Shinella zoogloeoides* DD12 | ESS77656 |
| 17 | 20451-20891 | → | 146 | hypothetical protein | 36%  (49/135) | *Rhizobium* sp. AP16 | WP_007699265 |
| 18 | 21056-21580 | → | 174 | transposase | 90%  (132/147) | *Ochrobactrum* sp. EGD-AQ16 | WP_021586543 |
| 19 | 21840-25652 | → | 1270 | outer membrane autotransporter barrel domain-containing protein | 93%  (765/820) | *O. anthropi* | WP_010657928 |
| 20 | 25803-26321 | ← | 172 | transposase | 99%  (170/172) | *O. anthropi* ATCC 49188 (plasmid pOANT01) | YP_001373005 |
| 21 | 26386-26640 | ← | 84 | transposase | 100%  (84/84) | *O. anthropi* ATCC 49188 (plasmid pOANT01) | YP_001373006 |
| 22 | 26795-27097 | ← | 100 | transposase | 76%  (65/86) | *O. intermedium* | WP_006469790 |
| 23 | 27426-28199 | ← | 257 | hypothetical protein | no similarity found | | |
| 24 | 28437-29258 | → | 273 | hypothetical protein | no similarity found | | |
| 25 | 29271-30206 | ← | 311 | hypothetical protein | 48%  (146/306) | *Ochrobactrum* sp. EGD-AQ16 | WP_021587220 |
| 26 | 30539-30922 | ← | 127 | 5'-nucleotidase | 56%  (78/139) | *Agrobacterium* sp. H13-3 | YP_004444282 |
| 27 | 30943-31260 | ← | 105 | hypothetical protein | 68%  (67/99) | *Starkeya novella* DSM 506 | YP_003694425 |
| 28 | 32255-32617 | → | 120 | transposase | 83%  (99/120) | *Rhizobium tropici* CIAT 899 | YP_007333345 |
| 29 | 32644-33201 | → | 185 | transposase | 98%  (181/185) | *Brucella* sp. BO2 | WP_009363880 |
| 30 | 33214-33753 | ← | 179 | transposase | 71%  (78/110) | *Brucella abortus* | WP_016650888 |
| 31 | 33902-34105 | ← | 67 | hypothetical protein | no similarity found | | |
| 32 | 34153-34581 | → | 142 | hypothetical protein | no similarity found | | |
| 33 | 34706-35098 | → | 130 | hypothetical protein | no similarity found | | |
| 34 | 35091-35669 | → | 192 | hypothetical protein/succinoglycan biosynthesis protein | 62%  (111/180) | *Agrobacterium radiobacter* K84 (plasmid pAtK84c) | YP_002546596 |
| 35 | 36071-37195 | ← | 374 | porin, Omp2 | 76%  (296/387) | *O. intermedium* | WP_006470887 |
| 36 | 37552-38973 | → | 473 | transposase | 83%  (391/473) | *Rhizobium mesoamericanum* | WP_007539883 |
| 37 | 38980-39717 | ← | 245 | hypothetical protein | 80%  (196/245) | *Rhizobium* sp. | WP_004675918 |
| 38 | 40074-40829 | ← | 251 | hypothetical protein | 36%  (66/185) | *Methylobacterium populi* BJ001 (plasmid pMOOP02) | YP_001919452 |
| 39 | 41056-42300 | ← | 414 | hypothetical protein | 23%  (95/406) | *Chelativorans* sp. BNC1 | YP_665820 |
| 40 | 42255-42818 | ← | 187 | hypothetical protein | 34%  (56/167) | *Chelativorans* sp. BNC1 | YP_665820 |
| 41 | 44538-44918 | → | 126 | membrane protein | 70%  (83/119) | *Agrobacterium tumefaciens* | WP_003506640 |
| 42 | 45025-45192 | ← | 55 | hypothetical protein | no similarity found | | |
| 43 | 45629-46501 | ← | 290 | LysR family transcriptional regulator | 60%  (169/284) | *Novispirillum itersonii* | WP_019643501 |
| 44 | 46697-47170 | → | 157 | hypothetical protein | 60%  (94/156) | *Agrobacterium vitis* S4 | YP_002548757 |
| 45 | 47211-48413 | → | 400 | aspartate aminotransferase A | 77%  (306/399) | *A. vitis* S4 | YP_002548756 |
| 46 | 48429-49034 | → | 201 | lysine transporter (LysE) | 79%  (158/201) | *A. vitis* S4 | YP_002548755 |
| 47 | 49110-50864 | → | 584 | hypothetical protein/FAD dependent oxidoreductase | 52%  (302/586) | *A. vitis* S4 | YP_002548754 |
| 48 | 50870-51487 | → | 205 | GCN5-related N-acetyltransferase | 33%  (56/169) | *Marinomonas mediterranea* MMB-1 | YP_004312508 |
| 49 | 51681-52298 | ← | 205 | acyltransferase | 66%  (131/197) | *A. radiobacter* K84 (plasmid pAtK84c) | YP_002546597 |
| 50 | 52295-52780 | ← | 161 | hypothetical protein | 47%  (67/144) | *Rhizobium freirei* | WP_004129419 |
| 51 | 52812-53375 | ← | 187 | hypothetical protein | 43%  (80/184) | *Rhizobium* sp. IRBG74 (plasmid IRBL74_p) | YP_008634013 |
| 52 | 53491-54258 | → | 255 | lytic transglycosylase | 46%  (99/216) | *A. radiobacter* K84 (plasmid pAtK84c) | YP_002546600 |
| 53 | 54261-54575 | → | 104 | type IV secretion protein VirB2 | 42%  (41/97) | *R. freirei* | WP_004129414 |
| 54 | 54657-54857 | → | 66 | type IV secretion protein VirB3 | 38%  (27/71) | *Methylocystis* sp. SC2 | CCD32144 |
| 55 | 54867-57314 | → | 815 | type IV secretion protein VirB4 | 60%  (480/797) | *A. radiobacter* K84 (plasmid pAtK84c) | YP_002546603 |
| 56 | 57311-58069 | → | 252 | hypothetical protein | 43%  (90/211) | *R. freirei* | WP_004129407 |
| 57 | 58066-58785 | → | 239 | type IV secretion protein | 63%  (141/223) | *Microvirga* sp. WSM3557 | WP_009762579 |
| 58 | 58790-58993 | → | 67 | hypothetical protein | 47%  (23/49) | *Microvirga* sp. WSM3557 | WP_009762580 |
| 59 | 58981-60000 | → | 339 | type IV secretion protein VirB6 | 36%  (101/277) | *Microvirga* sp. WSM3557 | WP_009762569 |
| 60 | 59997-60812 | → | 271 | type IV secretion protein VirB8 | 64%  (151/237) | *R. freirei* | WP_004129404 |
| 61 | 60809-61561 | → | 250 | hypothetical protein | no similarity found | | |
| 62 | 61561-62397 | → | 278 | type IV secretion protein VirB9 | 61%  (141/231) | *A. radiobacter* K84 (plasmid pAtK84c) | YP_002546609 |
| 63 | 62394-63641 | → | 415 | type IV secretion protein VirB10 | 61%  (257/423) | *R. freirei* | WP_004129402 |
| 64 | 63598-64698 | → | 366 | type IV secretion protein VirB11 | 63%  (227/359) | *A. radiobacter* K84 (plasmid pAtK84c) | YP_002546611 |
| 65 | 64691-66511 | → | 606 | type IV secretion protein VirD4 | 56%  (318/568) | *A. radiobacter* K84 (plasmid pAtK84c) | YP_002546612 |
| 66 | 67466-68002 | → | 178 | single-stranded DNA-binding protein | 60%  (107/178) | *O. anthropi* | WP_010660249 |
| 67 | 68260-69678 | → | 472 | autotransporter adhesin (YadA-like) | 61%  (213/352) | *Pseudochrobactrum* sp. AO18b | WP_022709815 |
| 68 | 69605-70669 | → | 354 | invasion associated locus B (IalB) | 39%  (88/228) | *Pseudochrobactrum* sp. AO18b | WP_022709816 |
| 69 | 70681-71169 | → | 162 | hypothetical protein | 59%  (79/135) | *Pseudochrobactrum* sp. AO18b | WP_022709817 |
| 70 | 71166-71372 | → | 68 | hypothetical protein | no similarity found | | |
| 71 | 71382-71828 | → | 148 | hypothetical protein | no similarity found | | |
| 72 | 71899-72153 | → | 84 | antitoxin of toxin-antitoxin | 82%  (69/84) | *Xanthomonas* sp. SHU308 | WP_017914822 |
| 73 | 72150-72578 | → | 142 | toxin of toxin-antitoxin | 76%  (103/135) | *Verminephrobacter aporrectodeae* | WP_010100821 |
| 74 | 72701-73792 | → | 363 | hypothetical protein | 45%  (160/354) | *Xanthomonas vasicola* | WP_017115150 |
| 75 | 74552-75736 | → | 394 | Sel1 domain-containing protein | 40%  (138/346) | *Magnetococcus marinus* MC-1 | YP_865910 |
| 76 | 75760-83541 | → | 2593 | putative secreted adhesin | 41%  (964/2362) | *Rhizobium* sp. CF122 | WP_007791119 |
| 77 | 83654-84748 | → | 364 | peptidase A2 | 45%  (156/347) | *Phyllobacterium* sp. YR531 | WP_008126343 |
| 78 | 84849-87035 | → | 728 | HlyB family type I secretion system ABC transporter ATP-binding protein, peptidase C39 | 78%  (566/725) | *Rhizobium* sp. CCGE 510 | WP_007630394 |
| 79 | 87032-88522 | → | 496 | HlyD family type I secretion membrane fusion protein, hemolysin D | 64%  (303/474) | *Agrobacterium* sp. H13-3 | YP_004443145 |
| 80 | 89702-94003 | ← | 1433 | relaxase (TraA) | 36%  (352/967) | *Rhizobium freirei* | WP_004129369 |
| 81 | 94003-94428 | ← | 141 | mobilization protein (MobC) | 35%  (44/125) | *Microvirga* sp. WSM3557 | WP_009489776 |
| 82 | 94988-95392 | → | 134 | hypothetical protein | 40%  (20/50) | *Shinella zoogloeoides* | WP_023517306 |
| 83 | 95733-96029 | ← | 98 | antitoxin of toxin-antitoxin | 63%  (61/97) | *Bartonella schoenbuchensis* | WP_010703104 |
| 84 | 96031-96402 | ← | 123 | toxin of toxin-antitoxin | 68%  (76/112) | *Bartonella rattimassiliensis* | WP_007348018 |
| 85 | 96555-96767 | ← | 70 | hypothetical protein | no similarity found | | |
| 86 | 96826-97119 | ← | 97 | hypothetical protein | 42%  (32/76) | *Pseudochrobactrum* sp. AO18b | WP_022712249 |
| 87 | 97180-97422 | ← | 80 | hypothetical protein | 73%  (29/40) | *Rhizobium mesoamericanum* | WP_007539845 |
| 88 | 97450-98067 | ← | 205 | hypothetical protein | 35%  (52/147) | *Rhizobium etli* bv. mimosae str. Mim1 | YP_008368571 |
| 89 | 98758-99441 | → | 227 | putative ParA-like protein | 36%  (81/228) | *Shinella zoogloeoides* | WP_023516063 |
| 90 | 99532-99768 | → | 78 | hypothetical protein | 46%  (24/52) | *Brucella melitensis* bv. 1 str. 16M | NP_540592 |
| 91 | 100215-100424 | ← | 69 | hypothetical protein | no similarity found | | |
| 92 | 100490-103732 | ← | 1080 | outer membrane autotransporter barrel domain-containing protein | 64%  (327/513) | *O. anthropi* ATCC 49188 (plasmid pOANT01) | YP_001373023 |
| 93 | 103981-104319 | → | 112 | transposase | 63%  (70/111) | *Nitratireductor aquibiodomus* | WP_007009506 |
| 94 | 104363-104941 | → | 192 | transposase | 76%  (135/177) | *Labrenzia* sp. | WP_023001591 |
| 95 | 105223-105492 | ← | 89 | hypothetical protein | 59%  (41/70) | *A. radiobacter* K84 | YP_002541857 |
| 96 | 105492-105884 | ← | 130 | hypothetical protein | 42%  (39/92) | *R. mesoamericanum* | WP_007533892 |
| 97 | 106143-106358 | ← | 71 | hypothetical protein | no similarity found | | |
| 98 | 106422-106850 | ← | 142 | hypothetical protein | 46%  (39/84) | *Oligotropha carboxidovorans* OM5 | YP_004633949 |
| 99 | 107018-107446 | ← | 142 | toxin of toxin-antitoxin | 75%  (106/142) | *Ochrobactrum* sp. CDB2 | WP_007881422 |
| 100 | 107443-107751 | ← | 102 | antitoxin of toxin-antitoxin | 77%  (62/81) | *Ochrobactrum* sp. CDB2 | WP_007881421 |
