# Supplementary material for: Diversity and role of plasmids in adaptation of bacteria inhabiting the Lubin copper mine in Poland, an environment rich in heavy metals
Source: Front Microbiol. 2015 Mar 3;6:152. doi: 10.3389/fmicb.2015.00152 (PMC4447125; doi:10.3389/fmicb.2015.00152)
Supplement: Supplementary file 12 [file Table7.DOC]

**Table S7.** Genes located within plasmids pLM8P1 of *Pseudomonas* sp. LM8 and pLM12P1 of *Pseudomonas* sp. LM12.

| Gene no. | **Coding region**  **(bp)** | **Strand** | **Protein size (aa)** | **Possible function** | **Best BLAST hits** | | |
| --- | --- | --- | --- | --- | --- | --- | --- |
|  |  |  |  |  | **% identity (aa)** | **Organism** | **GenBank accession no.** |
| **Plasmid pLM8P1 (1679 bp)** | | | | | | | |
| 1 | 64-930 | → | 288 | replication initiator protein | 70%  (181/260) | *Pseudomonas* sp. S-47 (plasmid p47S) | YP_232791 |
| 2 | 1329-1679 | → | 144 | CopG family transcriptional regulator | 64%  (72/113) | *Pseudomonas* sp. S-47 (plasmid p47S) | YP_232790 |
| **Plasmid pLM12P1 (5089 bp)** | | | | | | | |
| 1 | 647-1498 | → | 283 | replication initiator protein | 61%  (140/228) | *Pseudomonas fluorescens*  (plasmid pRO1600) | AAA98313 |
| 2 | 2060-2449 | → | 164 | hypothetical protein | 75%  (92/122) | *Pseudomonas aeruginosa* PSE9 | ABR13532 |
| 3 | 2460-4457 | ← | 665 | mobilization protein A (MobA) | 71% (184/259) | *Acidithiobacillus thiooxidans* ATCC 19377 | ZP_09995493 |
| 4 | 4604-5089 | → | 161 | mobilization protein C (MobC) | 66%  (44/67) | *Pseudomonas syringae* pv. maculicola (plasmid pPMA4326C) | YP_025711 |
