# Supplementary material for: Diversity and role of plasmids in adaptation of bacteria inhabiting the Lubin copper mine in Poland, an environment rich in heavy metals
Source: Front Microbiol. 2015 Mar 3;6:152. doi: 10.3389/fmicb.2015.00152 (PMC4447125; doi:10.3389/fmicb.2015.00152)
Supplement: Supplementary file 13 [file Table8.DOC]

**Table S8. Heavy metals tolerance of wild type strains and their derivatives carrying analyzed heavy metal resistance modules.**

|  | **Cd (II) tolerance^a^** | | | **Co (II) tolerance^a^** | | | **Zn (II) tolerance^a^** | | | **As (III) tolerance^a^** | | **As (V) tolerance^a^** | | **Hg (II) tolerance^a^** | | |
| --- | --- | --- | --- | --- | --- | --- | --- | --- | --- | --- | --- | --- | --- | --- | --- | --- |
|  | **wild type** | **CZC-**  **LM20^b^** | **CZC-**  **IS^c^** | **wild type** | **CZC-**  **LM20^b^** | **CZC-**  **IS^c^** | **wild type** | **CZC-**  **LM20^b^** | **CZC-**  **IS^c^** | **wild type** | **ARS-**  **LM20^d^** | **wild type** | **ARS-**  **LM20^d^** | **wild type** | **MER-**  **LM16^e^** | **MER-**  **TN^f^** |
| *Pseudomonas* sp. LM5 | 0.1 | **0.4** | 0.1 | 0.5 | **2** | 0.5 | 0.6 | **3** | 0.5 | 1 | **5** | 400 | 400 | 0.06 | **0.4** | 0.01 |
| *Pseudomonas* sp. LM6 | 0.1 | **0.6** | 0.1 | 0.5 | **2** | 0.4 | 0.6 | **2** | 0.5 | 0.6 | **3** | 75 | **600** | 0.2 | 0.09 | 0.08 |
| *Pseudomonas* sp. LM7 | 0.6 | 0.8 | 0.6 | 0.4 | 0.6 | 0.4 | 0.9 | 1.5 | 0.7 | 15 | 15 | 500 | 500 | 0.2 | **0.4** | 0.2 |
| *Pseudomonas* sp. LM8 | 0.1 | **0.3** | 0.1 | 0.5 | **1.5** | 0.3 | 0.4 | **1.5** | 0.4 | 7 | 7 | 250 | 250 | 0.2 | 0.15 | 0.25 |
| *Pseudomonas* sp. LM10 | 6 | 5 | 6 | 2 | 2 | 2 | 15 | 7 | 15 | 4 | 4 | 250 | 400 | 0.2 | 0.15 | 0.2 |
| *Pseudomonas* sp. LM12 | 0.2 | 0.08 | 0.1 | 0.6 | 0.7 | 0.6 | 0.8 | 0.9 | 0.7 | 2 | **5** | 400 | 400 | 0.2 | **0.4** | 0.06 |
| *Pseudomonas* sp. LM14 | 0.1 | 0.07 | 0.1 | 1.5 | 0.7 | 1 | 7 | 0.8 | 6 | 15 | 5 | 250 | 250 | 0.1 | **0.4** | 0.01 |
| *Pseudomonas* sp. LM15 | 1 | 1 | 0.9 | 4 | 4 | 3 | 7 | 6 | 6 | 7 | 7 | 500 | 350 | 0.01 | **0.4** | **0.04** |
| *Pseudomonas* sp. LM25 | 4 | 0.6 | 3 | 3 | 0.8 | 3 | 6 | 5 | 5 | 6 | 6 | 500 | 200 | 0.01 | **0.3** | **0.04** |
| *Achromobacter* sp.  LM16 | 7 | 2 | 6 | 1.5 | 1.5 | 1 | 10 | 7 | 10 | 25 | 25 | 1200 | 1200 | 0.3 | 0.15 | 0.07 |
| *Ochrobactrum* sp. LM19 | 2 | 2 | 1 | 1 | 1.5 | 1 | 10 | 7 | 9 | 9 | 9 | 1000 | 1000 | 0.02 | **0.3** | 0.03 |
| *Sinorhizobium* sp. LM21 | 2 | 0.4 | 1 | 1.5 | 0.9 | 1 | 3 | 3 | 3 | 5 | 6 | 200 | **600** | 0.02 | **0.3** | 0.03 |
| *Stenotrophomonas* sp. LM24 | 2 | 0.5 | 2 | 0.9 | 1.5 | 0.8 | 10 | 10 | 10 | 4 | 5 | 350 | 350 | 0.1 | **0.2** | 0.1 |
| *A. tumefaciens* LBA288 | 0.7 | **1.5** | 0.6 | 0.9 | 1 | 0.7 | 2 | **5** | 1 | 1 | **4** | 300 | 300 | 0.04 | **0.3** | 0.05 |
| *E. coli* TG1 | 3 | 3 | 2 | 0.5 | 0.4 | 0.3 | 3 | 3 | 2 | 3 | 3 | 100 | 100 | 0.09 | **0.2** | 0.04 |

^a^ - in table the MIC values (in mmol) were presented. At least 2-fold Increased (comparing with the wild type strains) MICs of strains carrying analyzed heavy metal resistance module are bolded.

^b^ – cobalt, zinc and cadmium resistance module of pLM20P2.

^c^ – cobalt, zinc and cadmium resistance module of IS*Ppu12a*.

^d^ – arsenic resistance module of pLM20P1.

^e^ – mercury resistance module of plasmid pLM16A1.

^f^ – mercury resistance module of plasmid Tn*5563a*.
